# Breast cancer cells that preferentially metastasize to lung or bone are more glycolytic, synthesize serine at greater rates, and consume less ATP and NADPH than parent MDA-MB-231 cells

**DOI:** 10.1186/s40170-023-00303-5

**Published:** 2023-02-20

**Authors:** Mika B. Jekabsons, Mollie Merrell, Anna G. Skubiz, Noah Thornton, Sandra Milasta, Douglas Green, Taosheng Chen, Yan-Hong Wang, Bharathi Avula, Ikhlas A. Khan, Yu-Dong Zhou

**Affiliations:** 1 Department of Biology, University of Mississippi, University, MS 38677 USA; 2 Department of Immunology, St Jude Children’s Research Hospital, Memphis, TN 38105 USA; 3 Department of Chemical Biology and Therapeutics, St Jude Children’s Research Hospital, Memphis, TN 38105 USA; 4 National Center for Natural Products Research, School of Pharmacy, University of Mississippi, University, MS 38677 USA; 5 Department of Biomedical Sciences, School of Pharmacy, University of Mississippi, University, MS 38677 USA; 6 Department of Chemistry and Biochemistry, University of Mississippi, University, MS 38677 USA

## Abstract

Gene expression signatures associated with breast cancer metastases suggest that metabolic re-wiring is important for metastatic growth in lungs, bones, and other organs. However, since pathway fluxes depend on additional factors such as ATP demand, allosteric effects, and post-translational modification, flux analysis is necessary to conclusively establish phenotypes. In this study, the metabolic phenotypes of breast cancer cell lines with low (T47D) or high (MDA-MB-231) metastatic potential, as well as lung (LM)- and bone (BoM)-homing lines derived from MDA-MB-231 cells, were assessed by ^13^C metabolite labeling from [1,2-^13^C] glucose or [5-^13^C] glutamine and the rates of nutrient and oxygen consumption and lactate production. MDA-MB-231 and T47D cells produced 55 and 63%, respectively, of ATP from oxidative phosphorylation, whereas LM and BoM cells were more glycolytic, deriving only 20–25% of their ATP from mitochondria. ATP demand by BoM and LM cells was approximately half the rate of the parent cells. Of the anabolic fluxes assessed, nucleotide synthesis was the major ATP consumer for all cell lines. Glycolytic NADH production by LM cells exceeded the rate at which it could be oxidized by mitochondria, suggesting that the malate-aspartate shuttle was not involved in re-oxidation of these reducing equivalents. Serine synthesis was undetectable in MDA-MB-231 cells, whereas 3–5% of glucose was shunted to serine by LM and BoM lines. Proliferation rates of T47D, BoM, and LM lines tightly correlated with their respiration-normalized NADPH production rates. In contrast, MDA-MB-231 cells produced NADPH and GSH at higher rates, suggesting this line is more oxidatively stressed. Approximately half to two-thirds of NADPH produced by T47D, MDA-MB-231, and BoM cells was from the oxidative PPP, whereas the majority in LM cells was from the folate cycle. All four cell lines used the non-oxidative PPP to produce pentose phosphates, although this was most prominent for LM cells. Taken together, the metabolic phenotypes of LM and BoM lines differed from the parent line and from each other, supporting the metabolic re-wiring hypothesis as a feature of metastasis to lung and bone.

**Supplementary Information:**

The online version contains supplementary material available at 10.1186/s40170-023-00303-5.

## Introduction

Breast cancer metastasis to lungs, bones, liver, or brain involves transcriptional changes that favor tumor cell extravasation, survival, and proliferation in the unique microenvironments of these organs [1]. Metabolic reprogramming that facilitates utilization of the most abundant substrates, promotes metabolite availability to biosynthetic pathways, ensures redox homeostasis by balancing reactive oxygen species (ROS) production with supply of reducing equivalents for antioxidant defenses, and synthesizes sufficient ATP to support the energetic cost of proliferation are particularly important for adaptation to microenvironments differing in trophic factor, oxygen, and/or nutrient availability. Brain-homing tumor cells were found to be enriched in energy metabolism (glycolysis, the TCA cycle, fatty acid oxidation, and oxidative phosphorylation) and antioxidant defense (the oxidative pentose phosphate pathway, glutathione reductase and transferase, and catalase) proteins relative to the parent breast cancer cells [2]. Additionally, brain-homing cells were found to have a higher capacity to oxidize glutamine and branched-chain amino acids, the latter of which are particularly abundant in the brain, than parent MDA-MB-231 cells [3]. These brain-homing characteristics were associated with greater tumor growth within the brain microenvironment compared to a bone-homing metastatic line introduced into the mouse forebrain [2]. Metabolic signatures, or phenotypes, may thus offer insight into organ-specific metastases.

Evidence to date suggests that changes to glucose and glutamine metabolism in lung and bone metastasis facilitate proliferation in these unique microenvironments [4]. Lung-homing lines derived from MDA-MB-435 cells were found to overexpress peroxiredoxin 2, which conferred resistance to exogenous ROS and efficacy for metastasis to the lungs [5]. By extension, this implies greater supply of NADPH by pathways such as the oxidative PPP for maintenance of peroxiredoxin redox state. Lung and bone metastatic lines that overexpress PGC-1α exhibit elevated glycolytic and respiratory capacities [6], consume glutamine at higher rates, and may utilize oxidative phosphorylation to a greater extent than parent breast cancer cells [7]. A bone-homing variant of MDA-MB-231 cells had significantly greater levels of enzymes that synthesize serine, as well as the SLC1A4 serine transporter, the latter of which has been proposed to promote serine export, osteoclastogenesis, and bone resorption [8]. However, a more comprehensive assessment of metabolic phenotypes that includes energy metabolism, anabolic pathways, and total ATP synthesis and turnover, is required for a more complete understanding of how organ-specific metastases differ.

Many studies of metastasis-associated metabolic changes overlook potential changes in ATP and NADH supply and demand, the former of which is important for determining the glycolytic phenotype and the latter of which is important to assessing TCA cycle fluxes. Recent studies have highlighted the importance of quantifying glycolytic and oxidative ATP production to obtain a more useful “glycolytic index” that cannot be obtained from measures of extracellular acidification or lactate efflux rates alone [6, 9–11]. For this, it is necessary to supplement measurements of nutrient uptake, waste production, and stable isotope enrichment in different metabolites with mitochondrial respiration rates. The latter rate not only provides a means to assess ATP derived from oxidative phosphorylation, but also imposes an important constraint to inferring TCA cycle and glutaminolysis fluxes by isotopic labeling, and on the shuttling of cytoplasmic NADH into mitochondria. The goal of this study was to quantitate metabolic fluxes in breast cancer lines differing in their metastatic potential (ER-positive T47D cells and more metastatic, triple-negative MDA-MB-231 cells), and bone- (BoM) and lung (LM)-homing lines derived from MDA-MB-231 cells [12, 13] to gain insight into the metabolic phenotypes that characterize lung and bone metastasis. Reactions synthesizing ATP (glycolysis, oxidative phosphorylation), contributing to the cellular redox state (the pentose phosphate pathway, malic enzyme, GSH synthesis), producing precursors for proliferation (fatty acids, nucleotides, serine), and involved in 1C metabolism were determined by analysis of respiration rates, glucose and glutamine consumption, lactate and serine efflux, and ^13^C labeling of the latter two metabolites from [1,2-^13^C] glucose or [5-^13^C] glutamine.

## Methods

### Reagents

Glutamate pyruvate transaminase, glucose-6-phosphate dehydrogenase (G6PDH), lactate dehydrogenase, nicotinamide adenine dinucleotide phosphate (NADP(H)), nicotinamide adenine dinucleotide (NAD), adenosine triphosphate (ATP), and α-ketoglutarate were purchased from Calzyme (San Luis Obispo, CA). Hexokinase, microbial glutamate dehydrogenase, 3-nitrophenylhydrazine, N-(3-dimethylaminopropyl)-N’-ethylcarbodiimide, buthionine sulfoximine, C75, and CBR-5884 were purchased from Sigma (St. Louis, MO). Glutaminase was purchased from Megazyme (Chicago, IL). [5-^13^C] glutamine, [1,2-^13^C] glucose, and [3-^13^C] lactate were purchased from Cambridge Isotopes (Tewksbury, MA). 6-Aminoquinolyl-N-hydroxysuccinimidyl carbamate (AQC) was purchased from Toronto Research Chemicals (Toronto, Canada). Cell culture media, fetal bovine serum, and cell culture supplies were purchased from Fisher Scientific (Pittsburg, PA). Buffers and other general reagents were purchased from Sigma.

### Cell lines

MDA-MB-231 and T47D cells were purchased from American Type Culture Collection. Sub-clones of MDA-MB-231 cells that preferentially metastasize to lung (LM clone 4175) and bone (BoM clone 1833), developed by Massague’s lab [12, 13], were provided by Dr. Kounosuke Watabe (Wake Forest University). Cells were routinely cultured in RPMI-1640 media (Corning, Manassas, VA) supplemented with 10% vol/vol fetal bovine serum (FBS, Hyclone, UT) and 50 U/mL penicillin and 50 μg/mL streptomycin (Gibco, MA) at 37°C in a humidified 5% CO_2_ incubator. Cells were maintained in culture for no more than 20 passages, and typically split twice a week. One day prior to experiments, 0.9–1.1 × 10^6^ cells were seeded in 2-well Lab-Tek chambers (Nalge Nunc International, NY) and maintained at 37°C in a humidified 5% CO_2_ incubator.

### Experiments with [5-^13^C] glutamine

Cells were incubated in 2-well Lab-Tek chambers for 20–22 h in either standard cell culture media supplemented with 10% FBS and penicillin (50 U/mL)-streptomycin (10 μg/mL) or glutamine-free RPMI-1640 media (Corning, VA) supplemented with 1.5 mM [5-^13^C] glutamine, 10% FBS, and penicillin (50 U/mL)-streptomycin (10 μg/mL) at 37°C/5% CO_2_. The following day, cells were switched to an experimental buffer (Buffer A) containing 137 mM NaCl, 5 mM KCl, 20 mM TES, 1.3 mM CaCl_2_, 1.3 mM MgCl_2_, 1.2 mM Na_2_SO_4_, 0.4 mM KH_2_PO_4_, 0.2 mM NaHCO_3_, 5.5 mM glucose, ± 1.5 mM [5-^13^C] glutamine, and 0.3 % wt/vol fatty acid-free bovine serum albumin (BSA), pH 7.4 for 5 h at 37°C. Cells seeded in standard culture media were given Buffer A without glutamine, and those seeded in media supplemented with [5-^13^C] glutamine were maintained in Buffer A + 1.5 mM [5-^13^C] glutamine. The buffer in each well was replaced once approximately 2.5 h into the equilibration period. Respiration rate of each well was determined at 37°C in 2.1 mL fresh Buffer A ± 1.5 mM [5-^13^C] glutamine using a Clark-type micro-oxygen electrode (Microelectrodes, Inc., Bedford MA) fitted to the top of the Lab-Tek chamber with a custom lid and magnetic stirrer assembly as previously described [14]. Data were acquired over 45–60 min using a Powerlab 20 A/D unit and Lab Chart software (AD Instruments). Wells were rinsed once prior to sequential 60-min incubations at 37°C with 350 μL Buffer A ± 1.5 mM [5-^13^C] glutamine. Samples were centrifuged 5 min, 21,000×*g*, 4°C, and the supernatant stored at −20°C for enzymatic analysis of glucose, lactate, and glutamine concentrations and ^13^C lactate content by mass spectrometry. For experiments with MDA-MB-231 cells, small cell pellets were consistently observed after sample centrifugation; the pellets were washed in phosphate buffered saline (PBS) then solubilized in Buffer B (see below) for quantification of protein content to correct for this loss with each incubation period. Control chambers without cells were incubated in parallel to assess changes in buffer volume (typically 10–25 μL) over each interval. At the end of each experiment, cells were rinsed once with 1 ml PBS then solubilized for 15 min at 37°C with 500 μL 50 mM NaCl, 20 mM TES, 1% SDS, pH 7.3 (Buffer B). The extracts were vortexed 2–3 min then centrifuged 10 min, 21,000×*g*, 10°C, and the supernatants stored at −20°C for quantification of total protein content by bicinchoninic acid (BCA) assay.

### Experiments with [1,2-^13^C] glucose

Cells were cultured in 2-well Lab-Tek chambers for 20–22 h in standard cell culture media supplemented with 10% FBS and penicillin (50 U/mL)-streptomycin (10 μg/mL). The next day, cells were equilibrated for 5 h, 37°C in experimental buffer (Buffer C) containing 137 mM NaCl, 5 mM KCl, 20 mM TES, 1.3 mM CaCl_2_, 1.3 mM MgCl_2_, 1.2 mM Na_2_SO_4_, 0.4 mM KH_2_PO_4_, 0.2 mM NaHCO_3_, 5.5 mM [1,2-^13^C] glucose, 0.3% fatty acid-free BSA, pH 7.4. Fresh Buffer C was replaced approximately 2.5 h into the equilibration period. Respiration rate of each chamber was determined at 37°C in 2.1 mL fresh Buffer C as previously described. Wells were rinsed once prior to sequential 60-min incubations at 37°C with 350 μL Buffer C. Samples were centrifuged 5 min, 21,000×*g*, 4°C, and the supernatants stored at −20°C for enzymatic analysis of glucose and lactate, and ^13^C content in lactate and serine by mass spectrometry. MDA-MB-231 cell pellets from the samples were saved and assayed for protein content to correct for protein loss over each incubation period. Control chambers without cells were incubated in parallel to assess changes in buffer volume (typically 10–25 μL) over each interval. At the end of each experiment, cells were rinsed once with 1 mL PBS then solubilized for 15 min at 37°C with 500 μL Buffer B. The extracts were vortexed 2–3 min then centrifuged for 10 min at 21,000×*g* at 10°C, and the supernatants stored at −20°C for quantification of total protein content by BCA assay.

### Pharmacological experiments

Cells were pre-incubated in Buffer A with unlabeled 1.5 mM glutamine that was additionally supplemented with either 40 μM C75 (or equivalent volume of 100% ethanol vehicle) for 3–4 h to inhibit fatty acid synthesis, 40 μM CBR-5884 (or equivalent volume of DMSO vehicle) for 2.5 h to inhibit serine synthesis, or 50 μM fresh BSO (or equivalent volume of water vehicle) for 2 h to inhibit glutathione synthesis. Buffer samples were collected after two (C75, BSO) or three (CBR-5884) sequential 60-min incubations with 350 μL buffer ± inhibitor, centrifuged 5 min, 21,000×*g*, 4°C, and the supernatants assayed for glucose, glutamine, and/or lactate to assess the extent to which the rates of nutrient uptake or lactate production were affected. Parallel control chambers without cells were run to correct for evaporative water loss over each time period. Cells were solubilized in Buffer B for quantification of total protein by BCA assay.

### Enzymatic assays for extracellular nutrients and lactate

For glucose quantitation, samples (5 μL, run in triplicate) were added to 115 μL assay buffer containing 100 mM triethanolamine, 7 mM MgCl_2_, 2 mM ATP, 2 mM NADP, 1 U/mL glucose-6-phosphate dehydrogenase, 1 U/mL hexokinase, pH 7.3. After 10 min, fluorescence was quantitated with a Shimadzu RF-6000 spectrofluorophotometer (*λ*_ex_=341 nm, *λ*_em_=464 nm, excitation and emission slits 5 and 15 nm, respectively). Glucose standards (3.5, 4.0, 4.5, 5.0, 5.5, and 6.0 mM) prepared in Buffer A were run in parallel.

For lactate quantitation, samples (5 μL, run in triplicate) were added to 115 μL assay buffer containing 100 mM glycylglycine, 100 mM glutamate, 2 mM NAD, 1 U/mL lactate dehydrogenase, and 1 U/mL glutamate pyruvate transaminase, pH 8.5. After 40min, fluorescence was quantitated with a Shimadzu RF-6000 spectrofluorophotometer (*λ*_ex_=341 nm, *λ*_em_=464 nm, excitation and emission slits 15 and 20nm, respectively). Lactate standards (0, 0.5, 1.0, 1.5, 2.0, and 2.5 mM) prepared in Buffer A were run in parallel.

Glutamine concentrations were determined by a two-step enzymatic reaction. For the first reaction, samples (6 μL, run in duplicate) were incubated 45 min at 37°C in 34 μL assay buffer containing 60 mM sodium acetate, ± 1 U/mL glutaminase, pH 4.5. Reactions without glutaminase served as controls to quantitate background ammonia production, which was subtracted to obtain the glutamine-derived ammonia. The second reaction was initiated by adding 80 μL of 300 mM Tris, 10 mM 2-oxoglutarate, 240 μM NADPH, and 2 U/mL microbial L-glutamate dehydrogenase, pH 8.5 to the completed first reaction. After 60 min incubation, fluorescence was quantitated with a Shimadzu RF-6000 spectrofluorophotometer (*λ*_ex_=341 nm, *λ*_em_=464 nm, excitation and emission slits of 10nm). Glutamine standards (0, 0.2, 0.4, 0.8, 1.2, 1.6, and 2.0 mM) prepared in Buffer A were run in parallel.

### Lactate derivatization and analysis by mass spectrometry

The samples and lactate standards in Buffer A (14 μL) were extracted with 2 volumes 100% cold methanol, incubated for 1 h, −20°C, and then centrifuged at 21,000×*g*, 2°C for 10 min. Freshly prepared 50 mM N-(3-dimethylaminopropyl)-N’-ethylcarbodiimide (in 1.5% pyridine, 98.5% ethanol, 21 μL) and 140 mM 3-nitrophenylhydrazine (in 50% ethanol, 21 μL) were added to each supernatant and incubated for 2 h, 37°C. The derivatized samples (30 μL) were diluted in 970 μL 80% methanol in mass spec vials for analysis by liquid chromatography-triple quadrupole mass spectrometry (LC-TQ MS). Both unlabeled (M), ^13^C labeled (M1), and 66:1 M:M1 lactate standards were run to confirm the expected masses (224 Da for M, 225 Da for M1) and verify separation of these isotopologues.

The derivatized samples were separated on a Waters ACQUITY I-Class UPLC™ system including binary solvent manager, sample manager, and column manager connected to a Waters Xevo TQ-S triple quadrupole mass spectrometer (Waters Corp, Milford, MA, USA). The separation was carried out on a Waters Acquity UPLC™ BEH C18 column (50mm × 2.1mm i.d., 1.7 μm). The sample temperature and column temperature were maintained at 10 and 40 °C, respectively. The mobile phase consisted of water containing 0.1% formic acid (v/v) (A) and acetonitrile with 0.1% formic acid (B). The analysis was performed using the following gradient elution at a flow rate of 0.50 mL/min: 0–2.5 min, 5% B to 18% B; 2.5–3 min, 18% B to 100% B. Each run was followed by a 2.5-min wash with 100% B and an equilibration period of 2.5 min with the initial conditions. The strong and weak solutions used to wash the auto sampler were methanol/acetonitrile/isopropanol/water (25:25:25:25, v/v/v/v/v) and methanol/water (70:30, v/v), respectively. The injection volume was 1 μL. The UHPLC effluent was introduced into the Waters Xevo TQ-S mass spectrometer equipped with electrospray ionization in negative ion mode (ESI−) for quantification of the analytes. Detection was obtained by Multiple Reaction Monitoring (MRM) mode including two MRMs for confirmation of the analytes. The quantification of analytes 224, 225, 226, and 227 (the expected masses for M, M1, M2, and M3 lactate derivatized with 3-nitrophenylhydrazine, respectively; all had 1.84 min retention times) was acquired with transitions of deprotonated ion at *m/z* 224.04 → 152.06 for M, 225.04 → 152.06 for M1, 226.04 → 152.06 for M2, and 227.04 → 152.06 for M3 with dwell time of 20 ms at cone voltage 44 V and collision energy 14 eV for each transition. The ESI–MS/MS parameters were set as follows: capillary voltage, 1.20 kV; cone voltage, 44 V; source temperature, 150 °C; desolvation temperature, 600 °C; desolvation gas flow, 600 L/h, cone gas flow, 200 L/h. Nitrogen was used as desolvation and cone gas. Argon (99.99% purity) was introduced as the collision gas into the collision cell at a flow rate of 0.15 mL/min. Data acquisition was carried out by MassLynx 4.1 software and processed by TargetLynx (Waters Corp., Milford, MA, USA).

### Serine derivatization and analysis by mass spectrometry

The samples and serine standards in Buffer A (20 μL) were extracted with 10 μL of 0.6 M trichloroacetic Acid (TCA) for 30 min on ice and then centrifuged for 10 min, 21,000×*g*, at 4°C. The supernatant (12 μL) was neutralized with 36 μL of sodium borate (pH 9.3). Stock 10 mM AQC was prepared fresh in anhydrous acetonitrile by incubating for 10 min at 55°C. Primary and secondary amines in the neutralized samples were derivatized with 24 μL 10 mM 6-aminoquinolyl-N-hydroxysuccinimidyl carbamate (AQC) for 10 min at 55°C.

^13^C serine labeling was analyzed on a Waters ACQUITY I-Class UPLC™ system coupled with a Waters Xevo TQ-S triple quadrupole mass spectrometer (Waters Corp, Milford, MA, USA). A Waters Acquity UPLC BEH C18 column (50 mm × 2.1 mm I.D., 1.7 μm) was used with a mobile phase composed of water containing 0.1 % formic acid (A) and acetonitrile with 0.1% formic acid (B). Sample and column temperatures were maintained at 10 and 40 °C, respectively. The LC flow rate was 0.35 mL/min in a gradient elution as follows: 0–1.5 min, 2% B to 20% B; 1.5–2.0 min, 20% B to 100% B. Each run was followed by a 2-min wash with 100% B and an equilibration period of 3 min with the initial conditions. The wash and purge solvents used to wash the autosampler were methanol/acetonitrile/isopropanol/water (25:25:25:25, v/v/v/v) and methanol/water (50:50, v/v), respectively. The effluent is directly guided into Xevo TQ-S mass spectrometer equipped with electrospray ionization in a positive ion mode (ESI+). Multiple Reaction Monitoring (MRM) mode including two MRMs for each analyte were used for quantification and confirmation respectively. MRMs of analytes 276, 277, 278, and 279 (the expected masses for M, M1, M2, and M3 of serine derivatized with AQC respectively; all had 1.08 min retention times) were recorded. For quantification of serine AQC derivatives, MRM transitions of protonated ion use at *m/z* 276.05 > 171.06 for M, 277.05 > 171.06 for M1, 278.05 > 171.06 for M2, and 279.05 > 171.06 for M3. The dwell time was 6 ms for each transition at cone voltage 46 V and collision energy 46 eV. The ESI+ MS/MS parameters were set as follows: capillary voltage, 2.50 kV; cone voltage, 38 V; source temperature, 150 °C; desolvation temperature, 500 °C; desolvation gas flow, 1000 L/h, cone gas flow, 150 L/h. Nitrogen was used as desolvation and cone gas. Argon (99.99% purity) as the collision gas was introduced into the collision cell at a flow rate of 0.15 mL/min. Data acquisition was carried out with MassLynx 4.1 software (Waters Corp., Milford, MA, USA).

### Protein assay

Cell extracts in Buffer B were diluted 1:3 in water. The diluted samples (25 μL, run in duplicate) were added to 500 μL of BCA Reagent then incubated for 30 min at 60°C. Absorbance at a 562 nm was determined using an Ultrospec 3100pro spectrophotometer (Amersham Bioscience). Bovine serum albumin standards (0, 50, 100, 150, 200, 300, 400, and 500 μg/mL) dissolved in 1:3 diluted Buffer B were used to quantitate total protein.

### Modeling and flux analysis

**Fig. 1:**
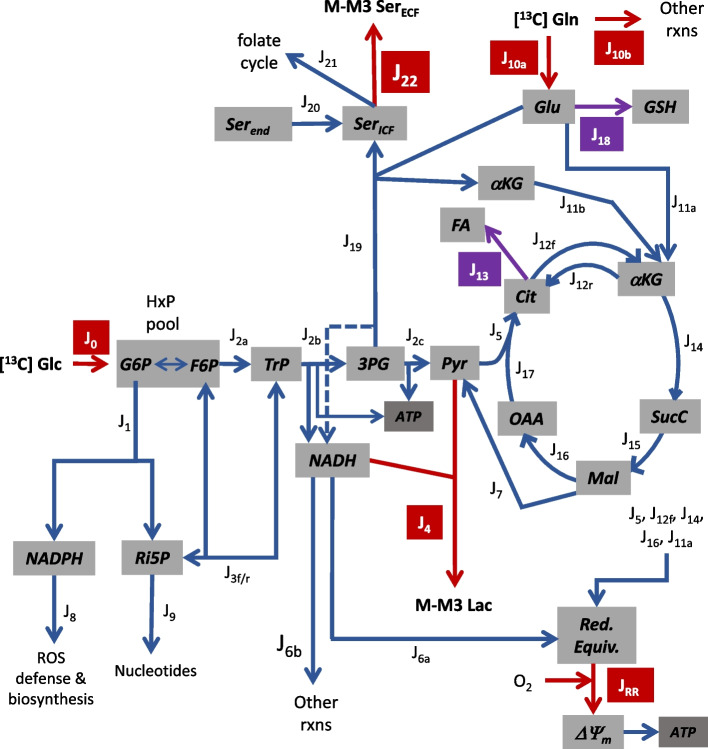
Tumor metabolism fluxes considered, except for those of the folate cycle. Red arrows: fluxes measured by extracellular substrate depletion (J_0_, J_10a+10b_, J_RR_) or product accumulation (J_4_, J_22_); purple arrows: fluxes measured by changes in glucose and/or glutamine consumption with pharmacological inhibitors (C75 for J_13_ and BSO for J_18_); blue arrows: inferred fluxes from ^13^C enrichment in lactate and/or serine (M-M3) from [1,2-^13^C] glucose or [5-^13^C] glutamine. Abbreviations: J_0_—glucose uptake/phosphorylation producing hexose phosphates (HxP), J_1_—oxidative pentose phosphate pathway (PPP) producing ribulose-5-phosphate (Ri5P) and NADPH, J_2a_—upper glycolysis producing triose phosphates (TrP: dihydroxyacetone phosphate and glyceraldehyde-3-phosphate), J_2b_—middle glycolysis producing 3-phosphoglycerate (3PG), ATP, and NADH, J_2c_—lower glycolysis producing pyruvate (Pyr) and ATP, J_3f_—forward non-oxidative PPP producing HxP and TrP, J_3r_—reverse non-oxidative PPP producing Ri5P, J_4_—lactate (Lac) production/export, J_5_—mitochondrial Pyr oxidation, J_6a_—mitochondrial oxidation of glycolytic NADH, J_6b_—oxidation of glycolytic NADH by other reactions, J_7_—cytoplasmic malic enzyme oxidation of malate (Mal), producing Pyr and NADPH (not shown), J_8_—NADPH consumption for ROS defense and biosynthesis, J_9_—Ri5P consumption for nucleotide synthesis, J_10a_—matrix glutamate (Glu) from glutamine (Gln) for glutathione (GSH) synthesis and glutamate dehydrogenase (GDH), J_10b_—Gln consumption by other reactions, J_11a_—GDH producing matrix α-ketoglutarate (αKG) and NADH, J_11b_—αKG from serine synthesis for the TCA cycle, J_12f_—citrate (Cit) oxidation and NADH/αKG production, J_12r_—NADPH-dependent reductive carboxylation of αKG for fatty acid (FA) synthesis, J_13_—FA synthesis from Cit, also yielding oxaloacetate (OAA; not shown), J_14_—αKG oxidation and NADH/succinyl CoA (SucC) production, J_15_—SucC oxidation yielding FADH_2_ and Mal, J_16_—Mal oxidation yielding NADH/OAA, J_17_—Cit synthesis from OAA and Pyr, J_18_—GSH synthesis from exogenous glutamine, J_19_—serine (Ser_ICF_) synthesis from 3PG, J_20_—endogenous serine (Ser_end_) from sources other than 3PG contributing to the Ser pool, J_21_—Ser for the folate cycle, J_22_—Ser export to the extracellular fluid (ECF), and Red. Equiv.—mitochondrial NADH/FADH_2_

Three models were developed using the solver function of Microsoft Excel to quantitatively track the relative ^13^C isotopologue abundance of key metabolites from the carbon atom rearrangements in glycolysis, the oxidative and non-oxidative pentose phosphate pathways (PPP), the tricarboxylic acid cycle, and the folate cycle to reproduce ^13^C labeling pattern in extracellular lactate and/or serine for cells consuming [1,2-^13^C] glucose or [5-^13^C] glutamine. Model 1 is based on the simplified metabolic scheme shown in Fig. 1 and predicts the fractional abundance of ^13^C lactate isotopologues (designated M, M1, M2, and M3 lactate, for zero, one, two, or three, respectively, ^13^C atoms) for cells metabolizing [1,2-^13^C] glucose as the sole exogenous substrate. Details of this model have been previously described [15, 16].

This model tracks carbon rearrangements as the atoms (a) recycle through the pentose cycle (the oxidative PPP and the forward non-oxidative PPP) to affect ^13^C labeling of the hexose phosphate (HxP) and triose phosphate (TrP) pools, (b) in HxP and TrP pools are metabolized through the reverse non-oxidative PPP to affect ^13^C labeling of the pentose phosphates, (c) in HxP pool are metabolized through glycolysis to affect ^13^C labeling of the TrP, 3-phosphoglycerate (3PG) and pyruvate pools, and (d) in pyruvate are metabolized through the tricarboxylic acid cycle and malic enzyme to affect ^13^C labeling of the malate and pyruvate pools, respectively. The measured variables for Model 1 were J_0_, J_4_, J_RR_, ^13^C lactate labeling, and C75-sensitive J_0_ and J_4_ (see Fig. 1 for flux abbreviations). The solver function optimized three flux ratios to minimize the difference (i.e., error) between the predicted and measured ^13^C lactate labeling pattern.

1 $$\frac{J_{3f}}{J_{3r}}=K$$

2 $$\frac{{}^{2}\!\left/ \!{}_{3}\right.\ast {J}_{3f}}{J_0+{}^{2}\!\left/ \!{}_{3}\right.\ast {J}_{3f}}=L$$

3 $$\frac{J_7}{J_{2c}+{J}_7}=M$$

The malate supplied for J_7_ was assumed to derive from matrix glutamate stores which equilibrate with the TCA cycle α-ketoglutarate pool; consequently, matrix reducing equivalents which contribute to respiration rate are generated in conjunction with J_7_, necessitating that malic enzyme flux indirectly contributes to J_RR_ and thus is constrained such that the model-predicted respiration rate equals the measured J_RR_. For some experiments where the model error was relatively high because measured J_RR_ was too low to adequately optimize Eq. 3 (primarily with LM cells), the fraction of glycolytic-derived NADH oxidized by mitochondria was also optimized.

4 $$\frac{J_{6a}}{J_{6a}+{J}_{6b}}=N$$

**Fig. 2:**
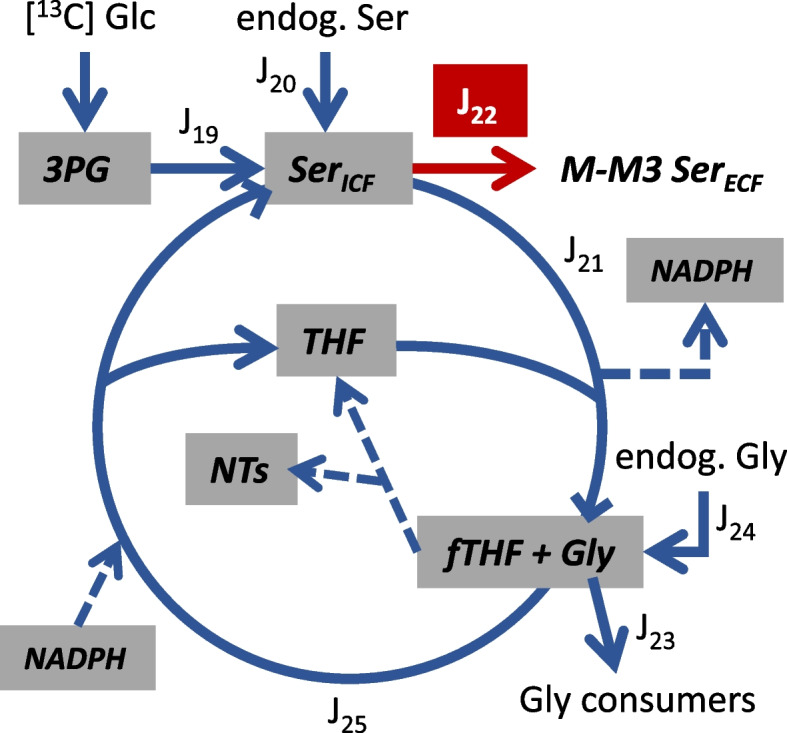
Simplified model of folate cycle fluxes. Red arrow reflects measured rate of extracellular (ECF) serine (Ser) accumulation (J_22_) and blue arrows are inferred fluxes from analysis of ^13^C serine enrichment (M-M3) from [1,2-^13^C] glucose. Abbreviations: J_19_—Ser synthesis from 3-phosphoglycerate (3PG), J_20_—the contribution of endogenous serine (Ser_end_) from sources other than 3PG to labeling the Ser pool, J_21_—mitochondrial Ser consumption by the folate cycle that produces glycine (Gly), NADPH, and 10-formyl tetrahydrofolate (fTHF), J_22_—Ser export to the ECF, J_23_—Gly consumption by reactions not producing Ser, J_24_—the contribution of endogenous Gly to labeling the Gly pool, and J_25_—Ser and tetrahydrofolate production from formate, Gly, and NADPH. Synthesis of purine nucleotides (NTs) recycles fTHF to THF without Ser production from Gly

Model 2 is based on the simplified metabolic scheme shown in Fig. 2 and predicts the fractional abundance of ^13^C serine isotopologues (designated M, M1, M2, and M3 serine, for zero, one, two, or three, respectively, ^13^C atoms) for cells metabolizing [1,2-^13^C] glucose as the sole exogenous substrate. This model uses ^13^C rearrangements from glucose to 3PG in Model 1 to track the probability of further carbon atom shuffling as serine recycles through the folate cycle.

A single cytoplasmic serine pool consisting of that enriched in ^13^C from 3PG and that from endogenous sources was considered as the substrate for the folate cycle. For serine entering the folate cycle (J_21_, Fig. 2), serine carbon 3 is transferred to tetrahydrofolate (THF), and the resulting glycine was assumed to enter a single pool along with endogenous glycine. As such, transfer of ^13^C from 10-formyl THF to endogenous glycine will result in M1 serine enrichment. The measured variables for Model 2 were J_22_, ^13^C serine labeling, and the endogenous, naturally occurring ^13^C labeling of serine and glycine (as measured with unlabeled serine standards). Knowledge of the positional ^13^C 3PG labeling (e.g., for M1, the proportion labeled at carbon 1, 2, or 3; for M2 the proportion labeled at carbons 1/2, 1/3, or 2/3) was also required, which was obtained from that predicted by Model 1. The solver function optimized the following three flux ratios to minimize the error between the predicted and measured ^13^C serine labeling pattern (see Fig. 2 for flux abbreviations).

5 $$\frac{J_{19}}{J_{19}+{J}_{20}}=P$$

6 $$\frac{J_{21}}{J_{21}+{J}_{24}}=Q$$

7 $$\frac{J_{25}}{J_{19}+{J}_{20}}=R$$

Model 3 is based on the simplified metabolic scheme shown in Fig. 1 and predicts the fractional abundance of ^13^C lactate isotopologues for cells metabolizing [5-^13^C] glutamine and unlabeled glucose as the two exogenous substrates. This model tracks carbon rearrangements from glutamine within the tricarboxylic acid cycle upon entry into the cycle as α-ketoglutarate to affect ^13^C malate labeling, and hence lactate labeling by J_7_. The measured variables for Model 3 were J_0_, J_4_, J_10_, J_RR_, ^13^C lactate labeling, C75-sensitive J_0_, J_4_, and J_10_, and BSO-sensitive J_10_. To solve for all fluxes (Figs. 1 and 2), additional inputs for Model 3 were Equations 1, 2, 5, 6, and 7 determined from Models 1 to 2. The Model 3 solver function optimized three flux ratios to minimize the difference (i.e., error) between the predicted and measured ^13^C lactate labeling pattern.

8 $$\frac{J_{11a}+{J}_{11b}}{J_{11a}+{J}_{11b}+{J}_{12f}}=W$$

9 $$\frac{J_{11a}+{J}_{11b}}{J_{10}}=X$$

10 $$\frac{J_7}{J_{2c}+{J}_7}=Y$$

The carbon flux into the TCA cycle to support J_7_ and J_12r_ were assumed to be derived exclusively from metabolism of exogenous glutamine via J_11a_ and/or J_11b_ to produce α-ketoglutarate. As with Model 1, matrix reducing equivalents generated in conjunction with J_7_ and J_12r_ necessitated that both fluxes indirectly contributed to J_RR_ and were thus constrained so that the model-predicted respiration rates equaled the measured rates. As with Model 1, experiments with LM cells resulted in relatively large Model 3 errors because of low respiration rates that limited optimization of Eq. 10; in these cases, the fraction of glycolytic-derived NADH oxidized by mitochondria was also optimized to lower the error.

11 $$\frac{J_{6a}}{J_{6a}+{J}_{6b}}=Z$$

Models 1 and 3 yielded redundant flux ratios (Eq. 3/10, and Eq. 4/11) that in some cases differed (see Tables 1 and 4). Since all fluxes reported here are for experiments with exogenous glucose and glutamine, and predicting ^13^C enrichment in multiple metabolites in Model 3 was a substantially simpler undertaking compared to Model 1, the results from Eq. 10 and 11 were used. Fluxes were calculated from the measured variables and optimized flux ratios assuming metabolic and isotopic steady states, the structure of the metabolic network as shown in Figs. 1 and 2, and the known stoichiometries of the reactions. Details on flux calculations are provided in Supplementary Information.

### Statistics

Fractional ^13^C isotopologue labeling of lactate and serine, model-optimized flux ratios, nutrient consumption rates, lactate production rates, and inferred fluxes were analyzed by one-way ANOVA (cell line as the main factor), with Tukey’s post hoc test to determine significant differences (significance level set as *p* < 0.05). The effects of glutamine or pharmacological inhibitor on rates of nutrient consumption, waste production, or protein content within a cell line were analyzed by paired *t*-test. Principal component analysis, regression analysis, and bi-plots were performed using GraphPad Prism to visualize the relationships between the variables and the cell lines.

## Results

### Experiments with [1,2-^13^C] glucose to assess glucose shunted to the pentose phosphate pathway and to serine

^13^C enrichment of lactate differed significantly between cell lines given [1,2-^13^C] glucose as the sole exogenous substrate (Table 1). MDA-MB-231 lactate labeling differed significantly from the less malignant T47D line, with the former having lower M, M1, and M3, and higher M2 content. There were no significant differences between LM and BoM labeling, which had similarities with both the T47D and MDA-MB-231 lines. They tended to have higher M3 and lower M lactate compared to T47D cells, but higher M1 + M3, and lower M2 lactate compared to MDA-MB-231 cells.

**Table 1:** ^13^C lactate labeling and model-optimized flux ratios with [1,2-^13^C] glucose

	Lactate ^13^C isotopologue content (%)				Modeled flux ratios (%)				
	M	M1	M2	M3	$\frac{\frac{2}{3}{J}_{3f}}{J_0+\frac{2}{3}{J}_{3f}}$ (R1)	$\frac{J_{3f}}{J_{3r}}$ (R2)	$\frac{J_7}{J_{2c}+{J}_7}$ (R3)	$\frac{J_{6a}}{J_{6a}+{J}_{6b}}$ (R4)	Model error (%)
T47D	50.02±0.13 ^a^ (50.08±0.08)	3.20±0.06 ^a^(3.29±0.11)	45.89±0.19 ^a^ (45.89±0.19)	0.89±0.04 ^a^ (0.74±0.02)	8.9±0.5 ^a^	64±6 ^a^	3.9±0.1 ^a^	81±16 ^a^	0.42±0.09
MDA231	49.30±0.20 ^b^ (49.30±0.20)	2.52±0.07 ^b^ (2.35±0.08)	47.49±0.20 ^b^ (47.49±0.20)	0.69±0.06 ^b^ (0.85±0.04)	3.2±0.4 ^b^	40±380 ^a,b^	5.1±0.3 ^b^	100±0 ^a^	0.33±0.06
BoM	49.04±0.17 ^b^ (49.15±0.08)	3.28±0.14 ^a^ (3.23±0.24)	46.59±0.33 ^a^ (46.59±0.33)	1.09±0.05 ^a,c^ (1.03±0.05)	5.5±0.7 ^c^	30±43 ^a,b^	5.9±0.3 ^b^	83±17 ^a^	0.37±0.19
LM	49.08±0.05 ^b^ (49.25±0.13)	3.03±0.05 ^a^ (3.18±0.13)	46.60±0.12 ^a^ (46.60±0.12)	1.29±0.05 ^c^ (0.97±0.07)	6.2±0.2 ^a,c^	30±3 ^b^	5.4±0.4 ^b^	17±9 ^b^	0.73±0.22

Cells were equilibrated in 2-well Lab-Tek chambers for approximately 6.5 h with 5.5 mM [1,2-^13^C] glucose as the sole exogenous substrate. Basal respiration rate was assessed from 5 to 6.5 h, followed by 2 × 1 h incubations to determine the rates of glucose uptake, lactate and serine efflux, and ^13^C enrichment in lactate and serine. Respiration rates averaged 3.7±0.2, 25±4, 15±3, and 4.3±0.5 nmol O/min × mg for T47D, MDA-MB-231, BoM, and LM cells, respectively (mean ± SEM, *n*=6). ^13^C lactate labeling (mean ± SEM, *n*=6) is expressed as percent contribution of each isotopologue to total extracellular lactate (M, M1, M2, and M3 denote lactate with none, one, two, two, or three ^13^C atoms, respectively). Data in parentheses are simulated lactate labeling (mean ± SEM, *n*=6) from Model 1-optimized flux ratios R1, R2, R3, and R4. Model error was calculated as the sum of the absolute values of the difference between the measured and simulated M-M3 lactate. Within a column, means sharing common superscripts do not significantly differ

Four pathways responsible for lactate labeling were considered—the pentose cycle’s contribution to labeling the HxP and TrP pools (Table 1, R1), the contribution of reverse flux through the non-oxidative PPP in labeling the Ri5P pool (Table 1, R2), malic enzyme’s contribution to labeling the pyruvate pool (Table 1, R3), and the fraction of glycolytic NADH oxidized by mitochondria (Table 1, R4). While the latter ratio does not directly affect lactate labeling, it can do so indirectly by restricting R3 when mitochondrial respiration rate is less than the rate of glycolytic NADH production not required for lactate synthesis. The fraction of glucose converted to fatty acids (measured as C75-sensitive glucose consumption; Supplemental Fig. 2), and the contribution of this to mitochondrial respiration rate, was accounted for, given that respiration rate is an important constraint for calculating fluxes that produce reducing equivalents that can be used by the respiratory chain.

**Fig. 3:**
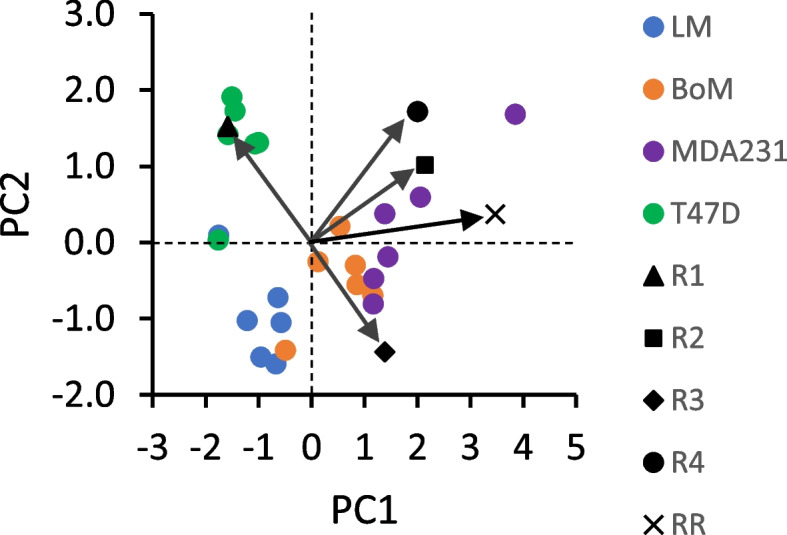
Bi-plot of Loadings and Scores from principal component analysis of Table 1 data. Flux ratios R1–R4 (see Table 1) were determined from Model 1 by simulating ^13^C lactate labeling from [1,2-^13^C] glucose, within the constraints of measured respiration rates (RR) and fluxes. The Scores reflecting individual experiments clustered into groups that corresponded to the four cell lines, with the less malignant T47D line more distant from the other three. The Loadings are indicated by arrows and provide information on relative strength of correlations between the variables R1–R4 and RR used to simulate ^13^C lactate labeling

Optimization of R1, R2, R3, and R4 by Model 1 (see Supplemental Information) yielded simulated labeling patterns similar to that measured by LC-TQ MS, but with errors primarily in M1 and M3 content (Table 1). Relative forward to reverse flux through the non-oxidative PPP (R2) was more variable than R1, R3, and R4 because relatively large changes to this ratio had only small effects on simulated lactate labeling compared to R1 and R3. Principal component analysis was performed to assess correlations among the variables (ratios R1–R4 and respiration rate, the latter of which was a constraint for R3 and R4) and clustering of the cell lines (Fig. 3). The first two principal components (PC1, PC2) accounted for 63.9% of variance in these ratios, and hence in lactate labeling. Respiration rate and the fraction of glycolytic NADH oxidized by mitochondria (R4) positively correlated. The relative non-oxidative PPP flux (R2) also correlated with respiration rate and R4, although this was strongly influenced by the large variance in R2 for the MDA-MB-231 cells. The contribution of the pentose cycle to labeling the HxP pool (R1) negatively correlated with malic enzyme labeling of the pyruvate pool (R3). The less malignant T47D cells formed a distinct cluster that was relatively far removed from the three malignant lines, largely because of greater pentose cycle activity. The three more malignant lines tended to form discrete clusters because of differences in respiration rate and the fraction of glycolytic NADH oxidized by mitochondria (Table 1, Fig. 3). It should be noted that M3 lactate simulated for LM cells exhibited the greatest error because R3, which is coupled to TCA cycle production of reducing equivalents, remained constrained by the low mitochondrial respiration rate (Table 1). Malic enzyme was a primary driver of lactate labeling for the three more malignant lines, but the LM cluster was more removed from those of the BoM and MDA-MB-231 cells because of their limited mitochondrial respiration and oxidation of glycolytic NADH (Table 1, Fig. 3).

**Fig. 4:**
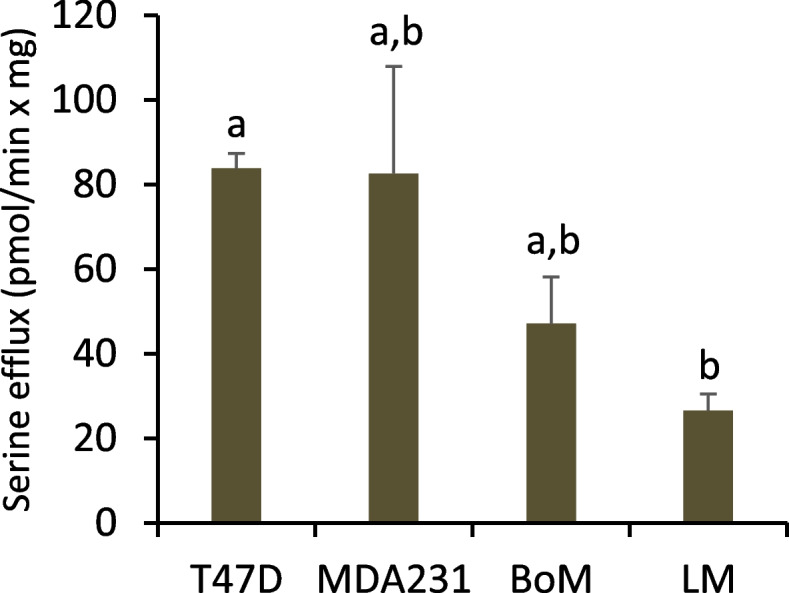
Serine efflux (J_22_) for cells metabolizing [1,2-^13^C] glucose. Serine (M-M3) was quantified by TQ-MS as detailed in the “Methods”. The efflux rate was calculated as the sum of all serine isotopologues in the ECF divided by 60-min incubation (averaged from two incubation periods). Data are mean ± SEM of six experiments for each cell line. Significant differences were assessed by one-way ANOVA with Tukey’s post hoc test. Bars sharing common letters are not significantly different

Serine efflux rate and ^13^C enrichment from [1,2-^13^C] glucose were assessed to estimate serine synthesis and cytoplasmic NADH production not coupled to pyruvate production. The rate of serine efflux tended to be greater in T47D and MDA-MB-231 cells (Fig. 4).

**Fig. 5:**
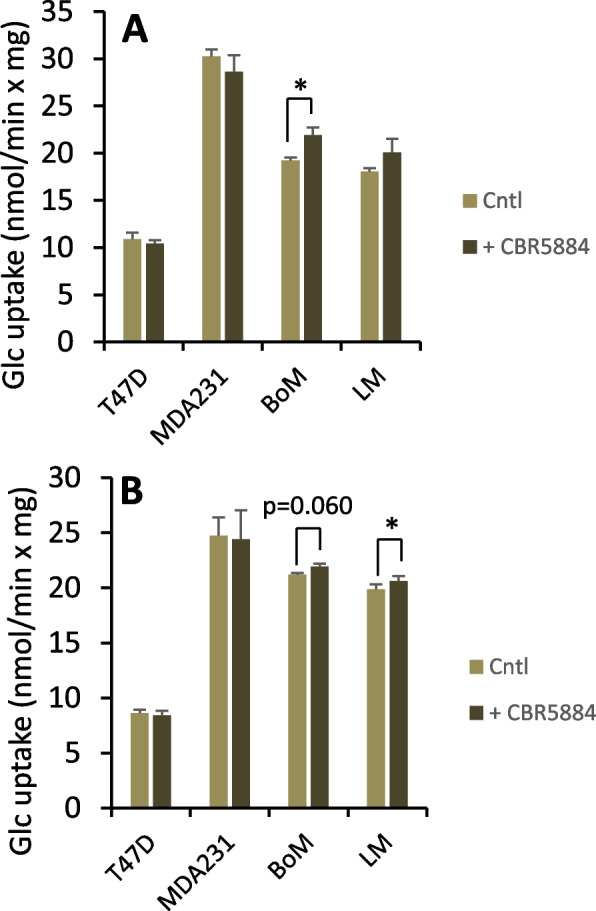
Glucose uptake rate by cells pretreated with the serine synthesis inhibitor CBR-5884. Cells were pretreated 2.5 h with 40 μM CBR-5884 or equal volume of DMSO in buffer without (**A**) or with (**B**) 1.5 mM glutamine. Average glucose uptake was determined over two 1-h intervals. Data are mean ± SEM of three experiments. * *p* < 0.05 by paired two-tailed *t*-test

Serine exported by MDA-MB-231 cells was not enriched above naturally occurring levels, while significant enrichment was observed for LM, BoM, and T47D cells, indicating the latter three lines synthesize serine from glucose (Table 2). The trend toward greater enrichment by LM and BoM cells suggest potentially higher serine synthesis in these lines. However, it was not possible to estimate glucose flux to serine by inhibition of serine synthesis, as CBR-5884 did not significantly reduce glucose uptake, but paradoxically stimulated uptake in LM and BoM cells while having no effect on T47D cells (Fig. 5).

**Table 2:** ^13^C serine labeling and model-optimized flux ratios with [1,2-^13^C] glucose

	Serine ^13^C isotopologue content (%)				Modeled flux ratios (%)			
	M	M1	M2	M3	$\frac{J_{19}}{J_{19}+{J}_{20}}$ (R5)	$\frac{J_{21}}{J_{21}+{J}_{24}}$ (R6)	$\frac{J_{25}}{J_{19}+{J}_{20}}$ (R7)	Model error (%)
T47D	82.21±0.65 ^a^ (82.21±0.65)	14.16±0.50 ^a^ (14.16±0.50)	3.46±0.19 ^a^ (3.46±0.19)	0.17±0.02 ^a^ (0.17±0.02)	29.9±2.9 ^a^	13.9±10.0 ^a^	11.0±0.3 ^a^	0.01±0.01
MDA231	95.25±0.37 ^b^ (nd)	3.88±0.29 ^b^ (nd)	0.84±0.07 ^b^ (nd)	0.03±0.04 ^b^ (nd)	0 ^b^	nd	nd	nd
BoM	67.93±4.73 ^a,c^ (67.93±4.73)	20.53±2.91 ^a^ (20.50±2.89)	11.04±1.74 ^c^ (11.04±1.74)	0.49±0.08 ^c^ (0.53±0.11)	69.0±14.6 ^a^	19.8±16.1 ^a^	6.6±0.3 ^b^	0.10±0.08
LM	66.58±2.69 ^c^ (66.58±2.69)	19.36±1.48 ^a^ (19.36±1.48)	13.43±1.16 ^c^ (13.43±1.16)	0.64±0.07 ^c^ (0.63±0.07)	63.9±10.4 ^a^	36.6±20.1 ^a^	5.6±0.2 ^c^	0.01±0.01

Experiments with [1,2-^13^C] glucose were conducted as detailed in Table 1. ^13^C serine labeling (mean ± SEM, *n*=6) is expressed as percent contribution of each isotopologue to total extracellular serine (M, M1, M2, and M3 denote serine with none, one, two, two, or three ^13^C atoms, respectively). Data in parentheses are simulated serine labeling (mean ± SEM, *n*=6) from Model 2-optimized flux ratios R5, R6, and R7. Model error was calculated as the sum of the absolute values of the difference between the measured and calculated M-M3 serine. Within a column, means sharing common superscripts do not significantly differ. Background ^13^C abundance in serine standards averaged 95.17±0.38, 4.06±0.33, 0.76±0.05, and 0.01±0.00 % for M, M1, M2, and M3 isotopologues, respectively. Note that the table reports total serine labeling not corrected for background. Nd: not determined

Given that carbon rearrangements do not occur as 3PG is converted to serine, it was surprising that the labeling pattern of 3PG (as predicted from Model 1) substantially differed from that of the exported serine (compare Tables 2 and 3). This indicated that once produced, serine carbons underwent rearrangement, likely through the folate cycle. With nearly all M2 3PG predicted to exist as C1C3 and C2C3 isotopomers, where C3 is transferred to tetrahydrofolate in the folate cycle, there is high probability that, when the cycle regenerates serine, the ^13^C initially removed will be transferred to unlabeled glycine from endogenous stores rather than the ^13^C glycine derived from serine (see Fig. 2). Thus, the cycle is predicted to enrich M1 serine at the expense of M2 serine.

**Table 3:** Simulated ^13^C 3-phosphoglycerate labeling with [1,2-^13^C] glucose

	Predicted 3PG ^13^C isotopologue content (%)			
	M	M1	M2	M3
T47D	51.31±0.08	2.07±0.11	46.51±0.19	0.11±0.01
MDA231	50.87±0.15	0.78±0.08	48.35±0.22	<0.01±0.00
BoM	51.03±0.14	1.35±0.16	47.58±0.30	0.05±0.02
LM	50.96±0.05	1.47±0.05	47.50±0.11	0.07±0.00

Experiments with [1,2-^13^C] glucose were conducted as detailed in Table 1. ^13^C 3-phosphoglycerate (3PG) labeling was determined from Model 1 results (Table 1). Relative isotopomer contents (not shown) were used to assess relative folate cycle fluxes (Table 2) to account for the difference between 3PG and serine labeling. Data are mean ± SEM of six experiments per cell line

**Fig. 6:**
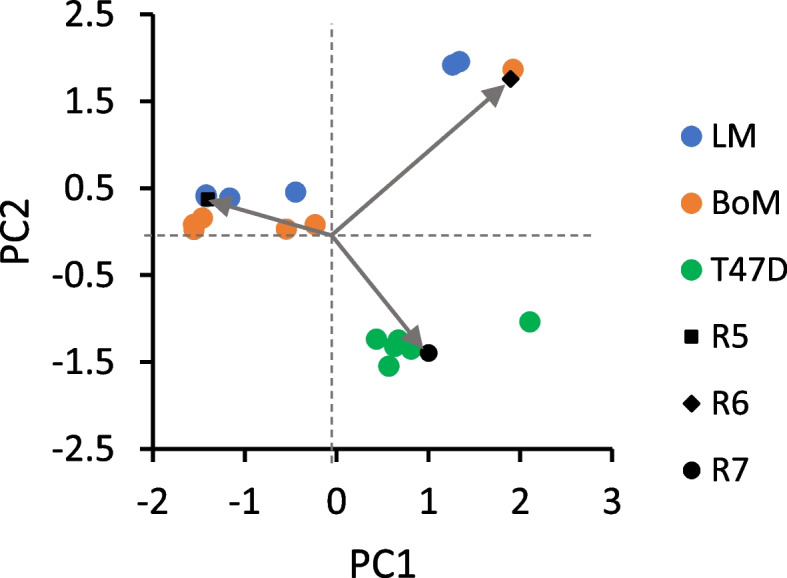
Bi-plot of Loadings and Scores from principal component analysis of Table 2 data. Flux ratios R5-R7 (see Table 2) were determined from Model 2 by simulating ^13^C serine labeling from [1,2-^13^C] glucose, using [^13^C] 3-phosphoglycerate labeling simulated by Model 1 (Table 3). The Scores reflecting individual experiments clustered into two groups—the less malignant T47D line and the LM/BoM lines, indicating differences in relative folate cycle fluxes for more vs. less malignant cells. MDA-MB-231 cells were not analyzed since there was no significant ^13^C enrichment in serine. The Loadings are indicated by arrows and provide information on relative strength of correlations between the variables R5–R7 used to simulate ^13^C serine labeling

A second model (Model 2) was developed to estimate three flux ratios associated with serine synthesis and the folate cycle to simulate the exported ^13^C serine labeling patterns: the contribution of serine synthesis vs. other endogenous serine sources to labeling the serine pool (Table 2, R5), the contribution of the folate cycle vs. other endogenous glycine sources to labeling the glycine pool (Table 2, R6), and the fraction of serine entering the folate cycle that recycles (Table 2, R7). Optimization of R5, R6, and R7 by Model 2 was sufficient to closely reproduce the measured labeling (Table 2) for those cell lines exhibiting significant ^13^C enrichment. From principal component analysis, PC1 and PC2 accounted for 94.7% of the variance in these ratios, and hence in serine labeling. Labeling of the serine pool by synthesis from 3PG (R5) negatively correlated with labeling of the same pool by the folate cycle (R7), whereas there was little correlation between folate cycle labeling of the glycine pool and R5 or R7 (Fig. 6). Consistent with the analysis of R1-R4 and respiration on ^13^C lactate labeling, the T47D cells formed a distinct cluster from the more malignant LM and BoM lines; this was primarily dictated by greater serine recycling in the folate cycle and less serine biosynthesis by T47D cells (Fig. 6).

### Experiments with [5-^13^C] glutamine to assess TCA cycle and malic enzyme fluxes

**Fig. 7:**
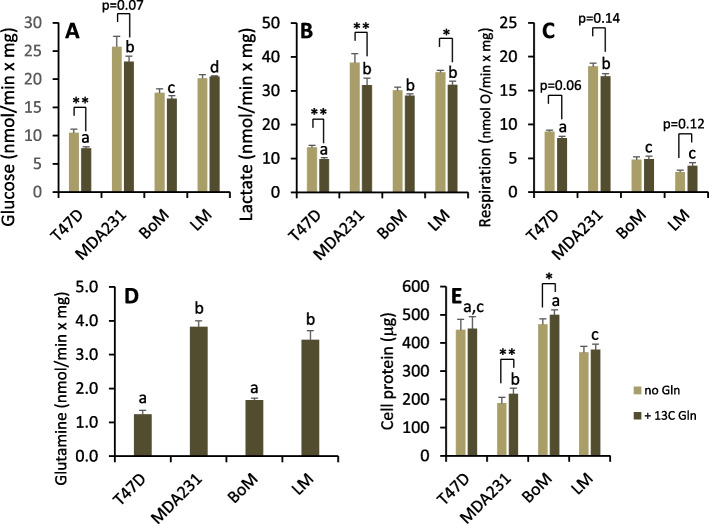
Substrate consumption, lactate production, and protein content for cells metabolizing glucose ± 1.5 mM [5-^13^C] glutamine. Glucose (**A**) and glutamine (**D**) depletion from the media and lactate accumulation (**B**) was determined over two 1-h intervals as detailed in the methods. Respiration rates (**C**) of these adherent cells was determined with a Clark-type micro-oxygen electrode as detailed in the “Methods”. SDS-soluble protein content was determined by BCA assay. Data are mean ± SEM of four experiments. Differences between cell lines metabolizing glucose + glutamine were analyzed by 1-way ANOVA with Tukey’s post hoc test. Bars sharing common letters are not significantly different. The effect of glutamine on each cell line was analyzed by paired, two-tailed *t*-test. * *p* < 0.05, ** *p* < 0.01

**Fig. 8:**
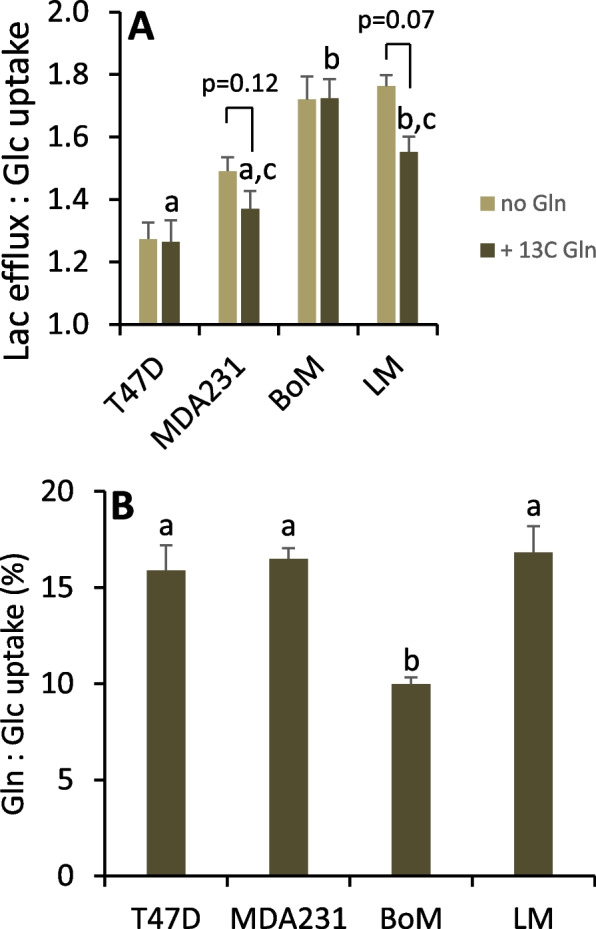
Indices of aerobic glycolysis ± glutamine (**A**) and relative glutamine-to-glucose preference (**B**). For cell lines metabolizing glucose + glutamine, bars sharing common letters are not significantly different, as determined by one-way ANOVA with Tukey’s post hoc test. The effect of glutamine on the ratio of lactate to glucose was determined by paired two-tailed *t*-test, with notable *p*-values indicated. Data are mean ± SEM of four experiments

The rates of glucose, glutamine, and oxygen consumption, and lactate production were determined for cells metabolizing unlabeled glucose and [5-^13^C] glutamine as exogenous substrates. Paired incubations without glutamine were run to assess background ^13^C lactate and the effect of glutamine on glucose metabolism and mitochondrial respiration. Glucose uptake and lactate production by MDA-MB-231, BoM, and LM cells were greater than T47D cells (Fig. 7A, B). A larger fraction of glucose used by LM and BoM cells was metabolized to lactate (Fig. 8A), and this was associated with lower rates of respiration by both of these cell lines (Fig. 7C). MDA-MB-231 and LM cells consumed glutamine at significantly greater rates than T47D and BoM cells (Fig. 7D). Glutamine tended to reduce glucose metabolism and respiration rate by T47D and MDA-MB-231 cells (Fig. 7A–C), whereas BoM cells were largely unresponsive to this substrate and exhibited the lowest preference for glutamine relative to glucose consumption (Fig. 8B). Glutamine tended to reduce aerobic glycolysis in LM cells given the trend to higher respiration rate and lower lactate-to-glucose ratio (Figs. 7C and 8A). Surprisingly, glutamine promoted MDA-MB-231 and BoM protein content over the relatively acute 10–11 h time course of these experiments (Fig. 7E).

**Fig. 9:**
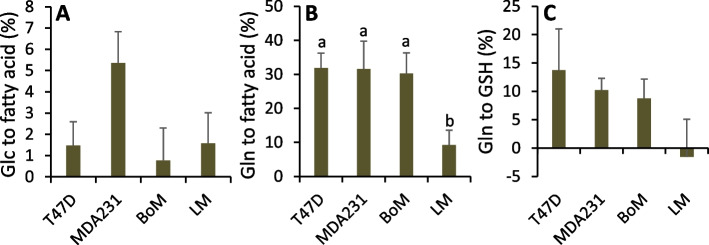
De novo synthesis of fatty acids and glutathione from glucose and/or glutamine. Cells were pretreated 3–4 h with 40 μM C75 to inhibit fatty acid synthesis or equivalent volume of vehicle (100% ethanol) (**A**, **B**). The decrease in glucose or glutamine consumption relative to controls was used to assess flux of these substrates to fatty acids as detailed in Supplemental Information Eq. 12 and 13. Cells were pretreated 2 h with 50 μM BSO to inhibit GSH synthesis or equivalent volume of vehicle (water) (**C**) and the decrease in glutamine uptake used to assess flux to GSH as detailed in Supplemental Information Eq. 11. Data are mean ± SEM of three to four experiments. Bars sharing common letters or having no letters do not significantly differ (1-way ANOVA *p*-values for effect of cell line: **A**, *p*=0.20; **B**, *p*=0.01; **C**, *p*=0.29)

Metabolism of glutamine to pyruvate and lactate was detected in all four cell lines by significant M1 lactate enrichment (Table 4). Glutamine seemed to be a particularly important mitochondrial substrate and precursor for malic enzyme in LM cells given M1 lactate enrichment was 2- to 5.8-fold greater than with the other cell lines and its tendency to enhance mitochondrial respiration (Fig. 7C). Consistent with this, LM cells shunted less glutamine to fatty acids and to glutathione compared to the other three lines (Fig. 9B, C), as estimated by inhibition of these anabolic pathways with 40 μM C75 or 50 μM BSO, respectively. Additionally, T47D, MDA-MB-231, and BoM cells significantly preferred glutamine over glucose as substrate for de novo fatty acid synthesis, whereas there was no such preference by LM cells (Fig. 9A vs. B).

**Table 4:** ^13^C lactate labeling and model-optimized flux ratios with [5-^13^C] glutamine

	Lactate ^13^C isotopologue content (%)				Modeled flux ratios (%)				
	M	M1	M2	M3	$\frac{J_{11a+b}}{J_{11a+b}+{J}_{12f}}$ (R8)	$\frac{J_{11a+b}}{J_{10}}$ (R9)	$\frac{J_7}{J_{2c}+{J}_7}$ (R10)	$\frac{J_{6a}}{J_{6a}+{J}_{6b}}$ (R11)	Model error (%)
T47D	99.13±0.09 ^a^ (99.15±0.08)	0.83±0.06 ^a^ (0.85±0.08)	0.03±0.01 ^a^ (0±0)	0.02±0.02 ^a^ (0±0)	46.6±1.3 ^a^	63.2±3.4 ^a^	3.5±0.2 ^a^	100±0 ^a^	0.11±0.04
MDA231	99.66±0.06 ^b^ (99.67±0.06)	0.33±0.06 ^b^ (0.33±0.06)	<0.01±0.01 ^a^ (0±0)	<0.01±0.00 ^a^ (0±0)	49.7±0.5 ^a^	43.5±1.6 ^b^	1.3±0.2 ^b^	100±0 ^a^	0.06±0.01
BoM	99.54±0.02 ^b^ (99.45±0.04)	0.46±0.02 ^b^ (0.55±0.04)	<0.01±0.00 ^a^ (0±0)	<0.01±0.00 ^a^ (0±0)	84.3±6.6 ^b^	52.8±3.0 ^a,b^	1.3±0.1 ^b^	95±5 ^a^	0.19±0.09
LM	98.05±0.06 ^c^ (98.11±0.07)	1.92±0.05 ^c^ (1.89±0.07)	0.02±0.00 ^a^ (0±0)	<0.01±0.00 ^a^ (0±0)	95.9±2.1 ^b^	47.4±4.1 ^b^	3.9±0.2 ^a^	0±0 ^b^	0.14±0.04

Cells were equilibrated with 1.5 mM [5-^13^C] glutamine and unlabeled glucose in 2-well Lab-Tek chambers for approximately 24–26 h. Basal respiration rate was assessed for approximately 1.5 h, followed by 2 × 1 h incubations to determine the rates of glucose and glutamine uptake, lactate production, and ^13^C enrichment in lactate. ^13^C lactate labeling (mean ± SEM, *n*=4) is expressed as percent contribution of each isotopologue to total extracellular lactate (M, M1, M2, and M3 denote lactate with none, one, two, two, or three ^13^C atoms, respectively). Data in parentheses are simulated lactate labeling (mean ± SEM, *n*=4) from Model 3-optimized flux ratios R8, R9, R10, and R11. Model error was calculated as the sum of the absolute values of the difference between the measured and calculated M-M3 lactate. Within a column, means sharing common superscripts do not significantly differ. Background isotopologue abundance for cells metabolizing unlabeled glucose averaged 96.52±0.05, 3.08±0.05, 0.40±0.01, and <0.01±0.01% for M, M1, M2, and M3, respectively. For these experiments, lactate labeling with [5-^13^C] glutamine was corrected for background labeling

**Fig. 10:**
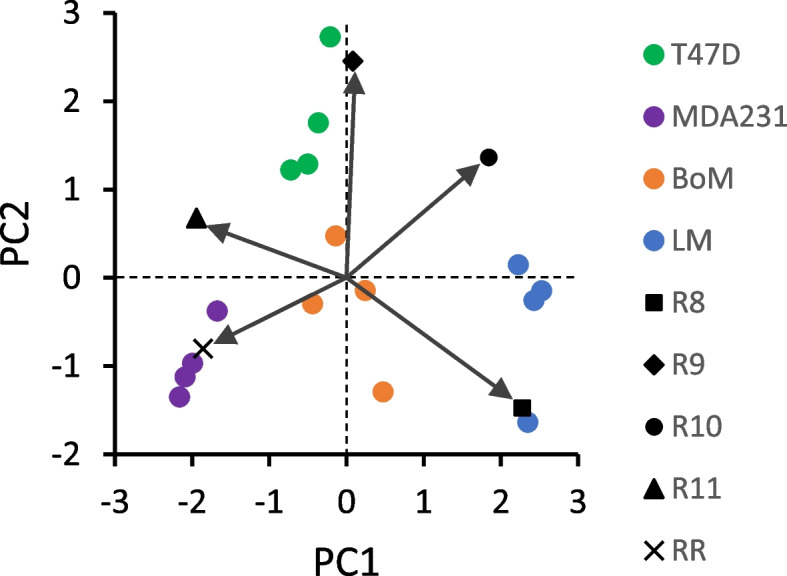
Bi-plot of Loadings and Scores from principal component analysis of Table 4 data. Flux ratios R8–R11 (see Table 4) were determined by Model 3 to simulate ^13^C lactate labeling from [5-^13^C] glutamine. The Scores from all experiments clustered into distinct groups by cell line. The arrows reflect the Loadings and show the correlations among the variables R8–R11 and respiration rate (RR) used to simulate ^13^C lactate labeling

A third model (Model 3) was developed to estimate relative fluxes producing or consuming key metabolites associated with the TCA cycle: the contribution of [5-^13^C] glutamine to labeling the α-ketoglutarate pool (Table 4, R8), the fraction of [5-^13^C] glutamine converted to α-ketoglutarate (Table 4, R9), the contribution of malic enzyme to labeling the cytoplasmic pyruvate pool (Table 4, R10), and the fraction of glycolytic-derived NADH oxidized by mitochondria (Table 4, R11). Optimization of R8-R11 by Model 3 was sufficient to closely reproduce ^13^C lactate labeling (Table 4). From principal component analysis, PC1 and PC2 accounted for 83.6% of the variance in these ratios, and hence in lactate labeling. As expected, respiration rate and the fraction of glycolytic NADH oxidized by mitochondria (R11) positively correlated (Fig. 10). The relative contribution of [5-^13^C] glutamine to the TCA cycle α-ketoglutarate pool (R8) only weakly correlated with cataplerotic flux of malate to pyruvate (R10) because α-ketoglutarate shunted to fatty acids did not correlate with R8. Furthermore, R8 negatively correlated with mitochondrial oxidation of glycolytic NADH (R11), implicating anapleurotic glutamine flux as a potential inhibitor of the malate-aspartate shuttle for tumor cells having low mitochondrial activity. This TCA cycle-focused flux analysis yielded more distinct, non-overlapping cell line clusters compared to the pentose cycle/glycolysis/TCA cycle flux analysis with [1,2-^13^C] glucose (Fig. 3). This suggests either glutamine metabolism differs more than glucose metabolism between these lines or that the larger errors, and hence greater uncertainty, associated with Model 1 is a limitation to resolving the differences in glucose metabolism. Despite this, both Models 1 and 3 show that MDA-MB-231 cells cluster with mitochondrial activity (respiration rate and fraction of cytoplasmic NADH oxidized by mitochondria) while LM cells weakly cluster with malic enzyme and negatively cluster with mitochondrial activity, while BoM cells reside between these two lines.

**Fig. 11:**
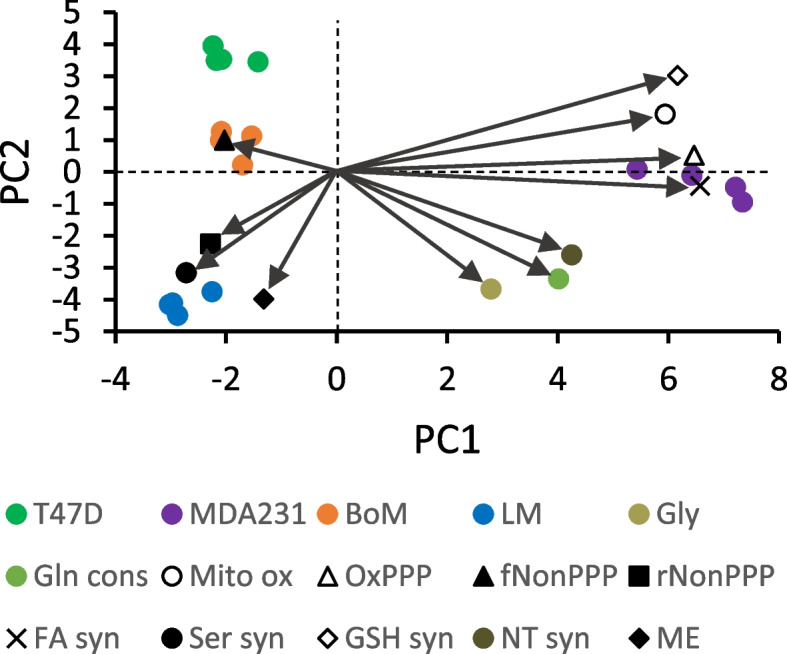
Bi-plot of Loadings and Scores from principal component analysis of Tables 5 and 6 data. To simplify the plot, dependent flux Loadings were consolidated. Abbreviations: Gly: glycolysis (average of J_2a_, J_2b_, J_2c_, J_4_); Gln cons: glutamine consumers (average of J_10a_, J_10b_); Mito ox: mitochondrial oxidation (J_5_, J_6a_, J_11a_, J_12f_, J_14_, J_16_, J_RR_); OxPPP: oxidative PPP (J_1_); fNonPPP: forward non-oxidative PPP consuming R5P (J_3f_); rNonPPP: reverse non-oxidative PPP producing R5P (J_3r_); FA syn: fatty acid synthesis (average of J_12r_, J_13_); Ser syn: serine synthesis (J_19_); GSH syn: glutathione synthesis (J_18_); NT syn: nucleotide synthesis (J_9_, J_21_); and ME: malic enzyme (J_7_)

The results from Models 1 to 3 (Tables 1, 2, and 4) were integrated with the measured fluxes from experiments with [5-^13^C] glutamine (Fig. 7), the pharmacological estimates of glutathione and fatty acid synthesis (Fig. 9), and the rate of serine export (Fig. 4) to infer fluxes through the reactions in Figs. 1 and 2 (Tables 5 and 6 and Fig. 11; see Supplemental Information for details on flux calculations). Glucose uptake differed significantly among the cell lines, but glycolysis (J_2a_, J_2b_, J_2c_) was similar for MDA-MB-231 and LM cells because of considerable glucose shunted to the PPP by the former line (Table 5). Glycolytic flux to pyruvate in BoM cells was significantly lower than MDA-MB-231 cells, yet lactate production did not differ because a higher proportion of pyruvate was consumed by mitochondria in the latter cell line. Thus, lactate efflux does not necessarily give an accurate measure of glycolytic flux (HxP to pyruvate). The pentose phosphate pathway was the second highest glucose consumer, but this was predominantly through the oxidative PPP for MDA-MB-231 cells (85% of R5P produced by J_1_) vs. the reverse non-oxidative PPP for LM cells (93% R5P from net J_3r_; Table 5 and Fig. 11). BoM and T47D cells produced R5P in a more balanced way. The fraction produced by the non-oxidative PPP significantly correlated with cell proliferation rate (Supplemental Fig. 3C). The percentage of glucose converted to R5P and nucleotides ranged from 9.5% by BoM cells to 26% by T47D cells, and negatively correlated with the glucose converted to lactate (R^2^=0.977), which was not surprising since it was assumed that glucose that was not (a) converted to lactate, (b) used for fatty acid and serine synthesis, or (c) oxidized by mitochondria was shunted to R5P for nucleotide synthesis.

**Table 5:** Glycolysis, pentose phosphate pathway, serine synthesis, and folate cycle fluxes

	Flux	T47D	MDA231	BoM	LM
	J_0_: Glc to HxP	7.78±0.26 ^a^	23.1±1.0 ^b^	16.6±0.5 ^c^	20.5±0.1 ^d^
Glycolysis	J_2a_: HxP to TrP	5.78±0.20 ^a^	17.8±1.1 ^b^	15.1±0.2 ^c^	17.4±0.4 ^b,c^
	J_2b_: TrP to 3PG	11.1±0.4 ^a^	35.4±2.2 ^b^	29.8±0.4 ^c^	33.5±0.9 ^b,c^
	J_2c_: 3PG to Pyr	10.6±0.5 ^a^	35.4±2.2 ^b^	28.8±0.4 ^c^	31.3±1.1 ^b,c^
	J_4_: Pyr to Lac	9.84±0.43 ^a^	31.7±2.0 ^b^	28.6±0.5 ^b^	31.8±1.1 ^b^
	J_6a_: NADH to Mitos	1.82±0.12 ^a^	3.70±0.17 ^b^	1.98±0.38 ^a^	0±0 ^c^
	J_6b_: NADH to Other	0±0 ^a^	0±0 ^a^	0.19±0.19 ^a^	3.86±0.45 ^b^
Ox and NonOx PPP	J_1_: HxP to R5P	1.12±0.15 ^a^	4.76±0.55 ^b^	0.81±0.22 ^a^	0.33±0.06 ^a^
	J_3f_: R5P to HxP/TrP	2.38±0.32 ^a^	0.53±0.06 ^b^	0.47±0.13 ^b^	1.78±0.17 ^a^
	J_3r_: HxP/TrP to R5P *	3.71±0.50 ^a^	1.35±0.16 ^b^	1.54±0.42 ^b^	5.85±0.56 ^c^
	Net J_3_: HxP/TrP to R5P *	1.33±0.18 ^a^	0.81±0.09 ^a^	1.07±0.29 ^a^	4.07±0.39 ^b^
	J_9_: R5P to NTs	2.45±0.33 ^a,c^	5.57±0.65 ^b^	1.89±0.51 ^a^	4.40±0.43 ^b,c^
Serine synthesis and folate cycle	J_19_: 3PG to Ser	0.54±0.07 ^a,b^	0±0 ^a^	1.00±0.27 ^b^	2.15±0.21 ^c^
	J_20_: Endog Ser to FC	1.27±0.17 ^a^	4.55±0.52 ^b^	0.45±0.12 ^a^	1.21±0.12 ^a^
	J_21_: Ser to Gly	1.96±0.27 ^a,c^	4.46±0.52 ^b^	1.51±0.41 ^a^	3.52±0.35 ^b,c^
	J_22_: Ser exported	0.06±0.00 ^a^	0.09±0.00 ^b^	0.04±0.00 ^c^	0.03±0.00 ^d^
	J_23_: Gly consumers	13.9±1.9	nd	7.52±0.2.05	9.43±0.93
	J_24_: Endog Gly to FC	12.1±1.6 ^a^	nd	6.11±1.66 ^b^	6.10±0.60 ^b^
	J_25_: Gly to Ser	0.20±0.03 ^a^	nd	0.10±0.03 ^b^	0.19±0.02 ^a,b^
	J_8_: NADPH consumers	4.37±0.56 ^a^	14.4±1.6 ^b^	3.41±0.79 ^a^	5.27±0.34 ^a^

**Fig. 12:**
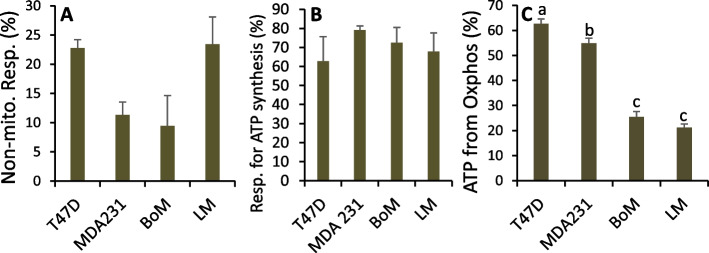
Estimates of ATP derived from mitochondrial oxidative phosphorylation. **A** Non-mitochondrial respiration rate was determined as the residual respiration rates measured by Seahorse instrument OCR after addition of 0.5 μM antimycin A and rotenone. Data are mean ± SEM of eight experiments. There was a significant main effect of cell line (1-way ANOVA *p* = 0.019), but no significant differences by Tukey’s post hoc test. **B** Oligomycin-sensitive OCR were measured by Seahorse instrument to estimate the percentage of mitochondrial respiration rate used for oxidative phosphorylation. Data are mean ± SD of two experiments. **C** Mitochondrial and glycolytic ATP synthesis rates for cells metabolizing unlabeled glucose + [5-^13^C] glutamine were calculated using Eq. 52 and 53 (Supplemental Information) and the percentage ATP derived from oxidative phosphorylation calculated. Data are mean ± SEM of four experiments. Bars sharing common letters are not significantly different

Data from Tables 1, 2, and 4 and Figs. 4, 7, 9, and 12 were used to calculate fluxes as detailed in Supplemental Information. Flux designations are as indicated in Figs. 1 and 2. Fluxes are in units of nmol substrate consumed per min per mg cell protein, except for those designated with * which are in units of nmol product (R5P) produced per min per mg cell protein. Data are mean ± SEM of four experiments. One-way ANOVA with Tukey’s post hoc test was used to determine significant differences. Within a row, means sharing common superscripts, or having no superscripts, do not significantly differ. Abbreviation: nd: not determined

**Table 6:** Glutamine, TCA cycle, malic enzyme, and GSH/fatty acid synthesis fluxes

	Flux	T47D	MDA231	BoM	LM
Gln and Glu metabolism	J_10a_: Gln to Glu	0.94±0.06 ^a^	2.04±0.06 ^b^	1.02±0.03 ^a^	1.60±0.08 ^c^
	J_10b_: Gln to Other	0.29±0.06 ^a^	1.78±0.14 ^b^	0.64±0.07 ^a^	1.84±0.30 ^b^
	J_11a_: αKG from GDH	0.77±0.05 ^a^	1.65±0.04 ^b^	0.87±0.03 ^a^	0.17±0.10 ^c^
	J_11b_: αKG from J_19_	0±0 ^a^	0±0 ^a^	0±0 ^a^	1.43±0.06 ^b^
	J_18_: Glu to GSH	0.17±0.02 ^a^	0.39±0.02 ^b^	0.15±0.01 ^a^	0±0 ^c^
TCA cycle, ME, and FA synthesis	J_5_: Pyr to Cit	1.11±0.04 ^a^	4.15±0.13 ^b^	0.53±0.07 ^c^	0.85±0.05 ^a^
	J_17_: OAA to Cit	1.11±0.04 ^a^	4.15±0.13 ^b^	0.53±0.07 ^c^	0.85±0.05 ^a^
	J_12f_: Cit to αKG	0.88±0.04 ^a^	1.67±0.05 ^b^	0.18±0.08 ^c^	0.08±0.04 ^c^
	J_12r_: αKG to Cit	0.39±0.04 ^a,c^	1.21±0.06 ^b^	0.50±0.02 ^a^	0.32±0.03 ^c^
	J_13_: Cit to FA	0.62±0.04 ^a^	3.68±0.15 ^b^	0.85±0.03 ^a,c^	1.09±0.02 ^c^
	J_14_: αKG to Suc	1.26±0.05 ^a^	2.12±0.05 ^b^	0.55±0.11 ^c^	1.36±0.13 ^a^
	J_15_: Suc to Mal	1.26±0.05 ^a^	2.12±0.05 ^b^	0.55±0.11 ^c^	1.36±0.13 ^a^
	J_16_: Mal to OAA	0.88±0.04 ^a^	1.67±0.05 ^b^	0.18±0.08 ^c^	0.08±0.04 ^c^
	J_7_: Mal to Pyr	0.38±0.02 ^a^	0.45±0.04 ^a^	0.37±0.04 ^a^	1.29±0.09 ^b^
	ATP consumers	21.7±1.0 ^a^	65.7±3.1 ^b^	36.1±0.9 ^c^	34.2±2.3 ^c^

Data from Tables 1, 2, and 4 and Figs. 4, 7, 9, and 12 were used to calculate fluxes as detailed in Supplemental Information. Flux designations are as indicated in Figs. 1 and 2. Fluxes are in units of nmol substrate consumed per min per mg cell protein. Data are mean ± SEM of four experiments. One-way ANOVA with Tukey’s post hoc test was used to determine significant differences. Within a row, means sharing common superscripts, or having no superscripts, do not significantly differ

Tricarboxylic acid cycle fluxes and mitochondrial respiration were highest for MDA-MB-231 cells and generally lowest for LM and BoM cells. Mitochondrial substrate oxidation by LM cells was particularly interesting in that the TCA cycle seemed to operate largely as a half-cycle from α-ketoglutarate to malate. Approximately 90% of pyruvate entering the cycle was used for fatty acid synthesis, leaving only 10% metabolized beyond citrate. Thus, 94% of TCA cycle α-ketoglutarate produced in LM cells was from glutamine rather than pyruvate oxidation; this primarily drove the half-cycle flux to malate in support of malic enzyme (Table 5 and Fig. 11). This scheme was inferred from the low respiration rate (Fig. 7C) and high ^13^C lactate enrichment from [5-^13^C] glutamine by LM cells (Table 4). It was only possible for Model 3 to simulate the measured ^13^C lactate labeling pattern within the constraint of the low respiration rate by assuming (a) none of the NADH generated by glycolysis was shuttled to mitochondria for oxidation (Table 5, J_6a_ = 0) and (b) virtually all of the α-ketoglutarate derived from glutamine was from a reaction not producing mitochondrial NADH (i.e., one that did not contribute to respiration rate). Serine biosynthesis was assumed as the source of α-ketoglutarate for the TCA cycle (Table 5, J_11b_) rather than glutamate/oxaloacetate transamination as oxaloacetate is likely required for oxidation of cytoplasmic NADH (see “Discussion”). The reliance of LM cells on glutamine to supply nearly all of the α-ketoglutarate in the TCA cycle, together with the fact that glutamine to malate flux accounted for 34% of matrix reducing equivalents for respiration rate is consistent with the finding that LM respiration rate tended to increase with exogenous glutamine (Fig. 7C). Conversely, exogenous glutamine, which tended to decrease MDA-MB-231 and T47D respiration rate, contributed only 20–30% to TCA cycle α-ketoglutarate supply and its flux to malate accounted for only 2.6 and 4.7 % of their respective respiration rates. Glutamine metabolism by BoM cells was more similar to LM cells in that it contributed approximately 67% of TCA cycle α-ketoglutarate, yet exogenous glutamine did not detectably stimulate respiration rate. For BoM cells, glutamine flux to malate could explain only 7.8% of respiration rate because of greater oxidation of pyruvate in the TCA cycle as well as the oxidation of cytoplasmic NADH.

The proportion of glucose used for serine and fatty acid synthesis was small relative to the glutamine used for fatty acid and GSH synthesis. Flux of 3PG to serine in LM cells was twice as great as in BoM cells (consuming 5.3 and 3.0% of glucose, respectively) and approximately fourfold greater than in T47D cells (consuming 3.5% of glucose), while no serine synthesis was detected in MDA-MB-231 cells (Tables 2 and 5, Fig. 11). Cell proliferation rate (Supplemental Fig. 3B) positively correlated with the fraction of glucose used for serine synthesis (Supplement Fig. 3C). Citrate flux to fatty acid was 3.4-fold higher for MDA-MB-231 than for LM cells, but both cell lines derived approximately 70% of de novo fatty acids from glucose vs. 30% from glutamine. Fatty acid synthesis by BoM and T47D cells was modestly lower than LM cells, and further differed from the other two lines in that the majority (approximately 60%) was derived from glutamine rather than glucose (40%). Cell proliferation rate negatively correlated with the fraction of glutamine diverted to fatty acids, whereas there was no significant correlation with the fraction of glucose diverted to fatty acids (Supplemental Fig. 3C). Glutathione, an important antioxidant, was synthesized by MDA-MB-231 cells at a significantly greater rate than T47D and BoM cells, although all three lines shunted about the same fraction of glutamine to GSH (8.8, 10.2, and 13.7 % for BoM, MDA2341, and T47D cells, respectively). Similarly, MDA-MB-231 cells synthesized NADPH, another important defense against ROS, at a significantly greater rate than the other cell lines (Table 5). Cell proliferation rate negatively correlated with the proportion of glutamine used for GSH synthesis (Supplemental Fig. 3C). Interestingly, the glutamine used for GSH and fatty acid synthesis correlated with the fraction of ATP derived from mitochondria (Supplemental Fig. 3A).

Non-mitochondrial respiration and the fraction of mitochondrial respiration for ATP synthesis were assessed as the myxothiazol-insensitive and oligomycin-sensitive respiration rates, respectively, by Seahorse analysis (Fig. 12A, B). From these data, and the stoichiometries of H^+^ pumped by the respiratory chain and H^+^ used by ATP synthase to synthesize ATP (see Supplemental Information), LM and BoM mitochondria were estimated to generate 21–25% of ATP, compared to 55–63% by MDA231 and T47D cells (Fig. 12C), confirming the highly glycolytic phenotype of the LM and BoM lines. Cell proliferation rates negatively correlated with the fraction of ATP from oxidative phosphorylation, but not with ATP demand (Supplemental Fig. 3C), which was highest for MDA-MB-231 cells (Table 6).

## Discussion

A main conclusion from this study is that two breast cancer lines previously [12, 13] selected for preferential metastasis to the lungs or bones have distinct metabolic phenotypes that likely reflect differences in their proliferation rates as well as their propensity for growth in the lung or bone microenvironments. Furthermore, each phenotype differed from that of the MDA-MB-231 cell line from which they were derived. The parent MDA-MB-231 cells consumed ATP at about twice the rate of the LM and BoM lines, relied approximately equally on oxidative phosphorylation and aerobic glycolysis to meet this ATP demand, and appeared to be more oxidatively stressed given their relatively high rates of NADPH consumption and GSH synthesis from glutamine. While LM and BoM lines were similar to each other in relying more heavily on glycolysis for ATP, LM cells exhibited a number of unique features, including (a) higher proliferation rate, (b) greater pentose phosphate consumption, (c) use of glutamine as a significant source of reducing equivalents for mitochondria, (d) use of the folate cycle as the primary source of NADPH, (e) use of the non-oxidative PPP as a major producer of pentose phosphates, and (f) limited shuttling of cytoplasmic NADH to mitochondria. The BoM line was distinguished by its low demand for pentose phosphates and NADPH, lower glutamine preference, low mitochondrial pyruvate oxidation and TCA cycle fluxes, and mitochondrial reducing equivalents largely supplied by glycolytic NADH. These results are consistent with metabolic reprogramming as a factor contributing to breast cancer metastasis and provide insights into the metabolic phenotypes that may confer survival and growth advantages in the lung and bone microenvironments.

Our results support and extend previous findings that MDA-MB-231 cells (a) have low expression of serine synthesis genes and hence little to no capacity to synthesize serine [8, 17–19], (b) have relatively active mitochondria (J_5_/J_4_ = 9.2% in [20] vs. 13.1% in this study), (c) consume glutamine at about 10–16% the rate of glucose (10.3% [20] vs. 16.5% in this study), (d) generate about 38–50% of TCA cycle α-ketoglutarate from glutamine (38.1% [20] vs. 49.7% in this study), (e) use 3–5% of glucose for fatty acid synthesis (3.6% [20] vs. 5.4% in this study), (f) synthesize 10–30% of de novo fatty acids from glutamine (~12% [21] vs. 30% in this study) by reductive carboxylation [20, 21], (g) shunt 20–25% of glucose through the oxidative PPP (25% in [20] vs. 21% in this study), (h) produce pyruvate by malic enzyme at 1–5% of the rate produced by glycolysis (5.6% in [20] vs. 1.3% in this study with [5-^13^C] glutamine), (i) have 2- to 4-fold greater rates of respiration, glucose uptake, and lactate production than the less metastatic T47D cells [22], and (j) exhibit a proliferation rate similar to the T47D cells [23]. Our results support previous findings that breast cancers with broad metastatic potential, including MDA-MB-231 cells, engage both high rates of glycolysis and oxidative phosphorylation [7]. The similarities with previous studies on MDA-MB-231 and T47D cells validate the current approaches to assessing fluxes through the major glucose- and glutamine-consuming reactions.

Our estimates of ATP turnover indicate MDA-MB-231 cells consumed ATP at a 3-fold higher rate than T47D cells despite similar rates of proliferation, and at a nearly twofold higher rate than BoM and LM cells despite higher proliferation rates by the latter two cell lines. More than half (55%) of ATP synthesized by MDA-MB-231 cells occurred by oxidative phosphorylation despite the relatively high rate of lactate production (Fig. 7, Table 5), and hence from this perspective, these cells were not “highly” glycolytic. This supports previous findings that 62% of ATP was from oxidative phosphorylation [24] and extends the conclusion that highly metastatic breast cancers exhibit both active glycolysis and oxidative phosphorylation [7]. This contrasts with lung and bone variants that synthesize only 21 and 25% of ATP, respectively, by oxidative phosphorylation. From the stoichiometries of ATP required by nucleotide, fatty acid, and GSH synthesis pathways, we estimated that collectively these pathways accounted for 92, 72, 43, and 102% of ATP consumption in T47D, MDA-MB-231, BoM, and LM lines, respectively. Of the ATP consumed by these anabolic pathways, >90% occurred through nucleotide synthesis for all four cell lines, highlighting the crucial energetic demand of this pathway in these tumor cells. It is notable that approximately 28 and 56% of ATP used by MDA-MB-231 and BoM cells, respectively, occurred through unidentified reactions (which equated to similar ATP turnover rates of 18.5 and 20.4 nmol/min × mg) compared to only 8 and −2% for T47D and LM cells, respectively. Since both MDA-MB-231 and BoM cells accrued significant new protein over the experimental time frame (Fig. 7E), this suggests that new protein synthesis accounted for at least some of their ATP consumption, despite the absence of exogenous essential amino acids in these experiments. Two other processes—maintenance of ionic gradients across the plasma membrane and oxidative stress—are suggested to also contribute to ATP consumed by MDA-MB-231 cells. Many MDA-MB-231 cells appear elongated, with a spindle-shaped morphology (Supplemental Fig. 4A–C) compared to the other three cell lines (T47D cells shown in Supplemental Fig. 4 D-F), indicative of a relatively high surface area-to-volume ratio. Additionally, MDA-MB-231 cells reach confluence at lower cell densities (~ 0.8–1.0 × 10^5^ cells/cm^2^ vs. 2.1–3.1 × 10^5^ cells/cm^2^ for T47D, LM, and BoM cells) and generally appear to form fewer close contacts with neighboring cells (Supplemental Fig. 4 A-C), the latter of which implies more membrane area exposed to the extracellular fluid. These factors may facilitate higher ion cycling rates across the plasma membrane, and hence greater ATP turnover to maintain these gradients. Oxidative stress, a potential feature of MDA-MB-231 cells (see below), promotes protein turnover that involves ATP-dependent degradation by the ubiquitin proteasome system that, together with new protein synthesis, yields an energy-consuming futile cycle that may also be responsible for the residual ATP use. Bone-homing cells did not exhibit spindle-shaped morphologies, nor did they appear to be oxidatively stressed, so pathways other than protein synthesis that may contribute to their residual ATP turnover are unknown. The considerable ATP turnover by MDA-MB-231 cells highlights their potential vulnerability to conditions that promote further ATP demand, particularly given previous findings of their relatively limited glycolytic and mitochondrial spare capacities [25]. Pharmacological therapies that facilitate ATP-dependent futile cycles and/or reduce the efficiency of ATP synthesis may thus exert more toxic effects on breast cancers with broad metastatic potential and high energy demand such as MDA-MB-231 cells.

Oxidative stress and diversion of resources for defense against ROS appears to be a feature of MDA-MB-231 cells given that GSH synthesis from glutamine and NADPH consumption are 2- to 4-fold higher than the other cell lines, and the fraction of glucose shunted through the oxidative PPP is greater (20.6% vs. 14.4%, 4.9%, and 1.6% for T47D, BoM, and LM, respectively). While NADPH is required for some anabolic reactions, MDA-MB-231 proliferation rate was lowest, suggesting the strong NADPH demand is more a function of oxidative stress than biosynthesis. Previous studies found that elevated expression of oxidative PPP and GSH-dependent enzymes in a breast cancer-derived brain metastatic clone paralleled upregulation of TCA cycle and oxidative phosphorylation components [2]; additionally, low ROS levels in a liver metastatic line was associated with low mitochondrial respiration [7], leading the authors to suggest that ROS production rates parallel mitochondrial activity. If true, then the ratio of NADPH demand to mitochondrial respiration rate can be taken as a pseudo ROS-normalized NADPH consumption for biosynthesis. Consistent with this possibility, proliferation rate of the cell lines significantly correlated with this ratio (Supplemental Fig. 3C). Notably, the MDA-MB-231 line was a relative outlier compared to the tight correlation (*R*^2^=0.997) for the other three lines (Supplemental Fig. 5), consuming NADPH at about a 1.7-fold greater rate than predicted by their proliferation rate from the T47D/BoM/LM correlation. This implies higher mitochondrial ROS production relative to their mitochondrial activity (i.e., respiration rate). As noted above, such an inference rests on the assumption that mitochondrial ROS production directly correlates with respiration rate, at least for T47D, BoM, and LM cells for the conditions used in this study. This may not be valid given that ROS production rates depend on a number of factors, including types of substrates available, the kinetics of the substrate oxidation, respiratory chain, ATP synthesis, and proton leak pathways, and the rate of ATP turnover [26]. Nonetheless, previous studies have demonstrated that, compared to luminal breast cancers such as T47D and MCF7 lines, MDA-MB-231 cells have higher ROS [27–29], greater GPX4, SOD1, or SOD2 expression [29, 30], higher protein carbonyls and lipid peroxidation products [29], and lower protein and non-protein thiols [27, 28], the latter of which may be a consequence of high H_2_O_2_ levels [31]. Taken together, the evidence implicates MDA-MB-231 cells as more oxidatively stressed and potentially vulnerable to redox active therapies in addition to those targeting ATP-dependent futile cycles.

Lung-homing LM cells lacked the capacity to shuttle cytoplasmic reducing equivalents into the matrix, minimally oxidized pyruvate through the TCA cycle, and correspondingly synthesized a minority (21%) of their ATP by oxidative phosphorylation. This glycolytic phenotype is similar to ErbB2 lung metastatic murine breast cancer cells that generate ~20% of ATP by oxidative phosphorylation [6]. Lack of a functional malate-aspartate shuttle could indicate dysfunctional mitochondria or a shift in metabolite or redox priorities. Malate import to the matrix as part of the shuttle system is coupled to export of α-ketoglutarate, yet two lines of evidence suggest the α-ketoglutarate carrier operates in reverse in LM cells (Table 6, J_11b_). First, there was considerable cytoplasmic α-ketoglutarate production coupled with serine synthesis (flux in excess of that required for malic enzyme), minimal matrix α-ketoglutarate produced from citrate, and relatively high cytoplasmic malate consumption by malic enzyme, all of which could make the metabolite gradients favorable for reverse transport by the α-ketoglutarate carrier. Second, the NADH produced in the cytoplasm beyond that required for lactate production, as well as the matrix NADH generated by glutamine metabolism to α-ketoglutarate (for malic enzyme), were well in excess of the measured mitochondrial respiration rates. The latter result made model simulations of LM ^13^C lactate labeling incompatible with the measured labeling patterns. Respiration rates and ^13^C lactate labeling were accurately simulated only when neither pathway was active (i.e., cytoplasmic NADH not shuttled into the matrix and glutamine not oxidized to α-ketoglutarate in the matrix). Thus, serine synthesis was considered as a source of α-ketoglutarate for the TCA cycle to support malic enzyme flux. Serine synthesis by triple-negative MDA-MB-468 cells was found to produce 50% of TCA cycle α-ketoglutarate [17]. Our results indicate that serine synthesis is even more important for the TCA cycle in LM cells, supplying approximately 94% of α-ketoglutarate used in the TCA cycle.

If limited respiration rate and reversal of the α-ketoglutarate transporter preclude normal operation of the malate-aspartate shuttle, then an alternative pathway is necessary for oxidation of cytoplasmic NADH (Table 5, J_6b_). Mitochondrial dysfunction in osteosarcoma cells caused by a mtDNA mutation resulted in loss of malate-aspartate shuttle activity and re-oxidation of NADH by cytoplasmic malate dehydrogenase 1 (MDH1) [32]. The reaction was sustained by malate export to the ECF, oxaloacetate supplied by ATP citrate lyase, and citrate from reductive carboxylation of α-ketoglutarate [32]. While the nature of low mitochondrial activity in LM cells is unknown, the deficit in shuttling cytoplasmic NADH into mitochondria raises the possibility that MDH1 is a candidate for J_6b_ (Fig. 1). Quantitatively, MDH1 activity must average 3.9 nmol oxaloacetate consumed/min × mg protein, yet the measured fatty acid and serine synthesis fluxes (the latter by transamination with aspartate) are predicted to supply only 47% of the required oxaloacetate. This implies an additional oxaloacetate producer that does not generate reducing equivalents is missing from the Fig. 1 model. Murine 4T1 cancer cells with a propensity for lung metastasis, as well as MDA-MB-231 cells, display significant pyruvate carboxylase expression and activity [33, 34] and hence this reaction could account for 2.1 nmol oxaloacetate/min required to re-oxidize the remaining NADH (see Supplemental Fig. 6 for proposed flux scheme for MDH1 as J_6b_).

A unique feature of the lung microenvironment is the ~3-fold greater P_O2_ compared to most systemic tissues [35]. In principle, this promotes ROS production due to a greater probability of electron leak from the respiratory chain to oxygen. Fluxes involved in antioxidant defenses were thus predicted to be greater for lung-homing cancers, yet LM cells surprisingly diverted no detectable exogenous glutamine toward GSH synthesis and appeared to primarily consume NADPH for biosynthesis to support their proliferation rate (Supplemental Fig. 5). While it is possible that LM cells synthesize GSH from internal glutamine/glutamate stores rather than from exogenous glutamine, the results suggest that they devote fewer resources to ROS defenses than the parent MDA-MB-231 line. As such, they may instead minimize ROS production in high oxygen environments such as the lungs by maintaining the respiratory chain in a more oxidized state by limiting mitochondrial import and/or oxidation of substrates. Evidence supporting this includes (a) limited respiration rate, (b) limited pyruvate dehydrogenase flux (J_5_), (c) 90% of acetyl CoA from pyruvate used for fatty acid synthesis rather than complete oxidation in the TCA cycle, and (d) no shuttling of cytoplasmic NADH to the matrix. This type of strategy is consistent with an increased reliance on aerobic glycolysis largely for ATP synthesis to compensate for limited oxidative phosphorylation. With potentially less resources required for antioxidant defenses, it is notable that the folate cycle accounted for the majority (63%) of NADPH production, while only 13% was from the oxidative PPP. Indeed, serine metabolism through the folate cycle accounted for 20–30% of NADPH production by HEK293T, MDA-MB-468, and iBMK cells, with >80% used for biosynthesis [36]. Limited oxidative PPP flux was inferred from R2 (Table 1, J_3f_/J_3r_; Eq. 30, Supplemental Information), which indicated more glucose shunted through the reverse non-oxidative PPP. Expression of the pyruvate kinase M2 isoform and the transketolase TKTL1 isoform have been associated with non-oxidative PPP flux [37], but allosteric regulation of the oxidative PPP by folate-derived NADPH likely has a role in limiting pentose phosphate production, thus necessitating a more prominent role for the non-oxidative PPP to support nucleotide synthesis. Indeed, the non-oxidative PPP was responsible for >90% of ribose phosphate production for de novo nucleotide synthesis to support the high proliferation rate of LM cells. This is similar to adenocarcinoma cells that were found to produce 85% of pentose phosphates through the non-oxidative PPP [38]. Metabolic re-wiring that limits the malate-aspartate shuttle and mitochondrial substrate oxidation, and promotes aerobic glycolysis, the re-oxidation of NADH by MDH1, folate cycle NADPH production, and pentose phosphate supply by the non-oxidative PPP may thus constitute a successful strategy for minimizing ROS production and supporting high proliferation rates in oxygen-rich environments.

Similar to the LM line, BoM cells primarily rely on glycolysis for their ATP and have low mitochondrial activity. Blood flow to bone is exceedingly low at 1–2 mL/min × 100 g [39]. After correction for hydroxyapatite content (approximately 65% of bone mass), blood flow (4–6 mL/min × 100 g non-mineral bone) is similar to that for skin (4–10 mL/min × 100 g), but considerably lower than highly metabolic or blood-servicing tissues such as exercising skeletal muscle (100–200 mL/min × 100g), brain (50–60 ml/min × 100 g), heart (70 mL/min × 100 g), kidneys (350 mL/min × 100 g), and lungs (300–500 mL/min × 100 g) [40]. The low perfusion-to-high bone marrow cell density is thought to create a relatively hypoxic bone environment, with P_O2_ ranging from 5 to 20 mmHg [41, 42]. Such hypoxia may explain previous findings that acute bone slice preparations exhibit a relatively pronounced glycolytic phenotype, metabolizing 72–80% of glucose to lactate [43, 44]. In this context, the strong aerobic glycolysis of BoM cells in vitro and lower glutamine-to-glucose preference is consistent with the more glycolytic phenotype of endogenous marrow cells that may be required for proliferation in the hypoxic marrow cavity. Thus, while BoM and LM cells have a similar glycolytic phenotype, this may reflect an adaptation by LM cells to limit mitochondrial ROS production in the hyperoxic lung environment whereas it may reflect an adaptation to a more hypoxic environment that limits mitochondrial activity for BoM cells.

Glutamine was not an important bioenergetic substrate for BoM cells given that it had virtually no effect on the rates of glucose uptake, lactate production, and mitochondrial respiration, whereas one or more of these fluxes were affected in the other cell lines (Fig. 7A–C). Consistent with this, BoM cells consumed less glutamine relative to glucose than the other three cell lines (Fig. 8B). In contrast, a higher glutamine-to-glucose preference has been reported in a bone-homing subclone compared to a parental 4T1 breast cancer line [7]. Glutamine preference may thus depend more on other specific metabolic signatures rather than the bone microenvironment. As noted earlier, glutamine positively affected BoM and MDA-MB-231 protein content over the acute time frame of these experiments (Fig. 7E) whereas it did not significantly affect T47D and LM cells. The pathways through which both glutamine and ATP were used may explain the different anabolic response to glutamine. The change in protein content among all cell lines positively correlated with the fraction of glutamine to fatty acids plus the undefined pathways not assessed in this study (*R*^2^=0.924; 78 and 69% of glutamine shunted for MDA-MB-231 and BoM cells, respectively vs. 63 and 56% for LM and T47D cells). Second, MDA-MB-231 and BoM cells used a larger fraction of ATP for pathways other than nucleotide, fatty acid, and GSH synthesis (28 and 56%, respectively, vs. 8 and −2% by T47D and LM cells, respectively). This suggests that at least some of the ATP available for “other” reactions was devoted to protein synthesis.

Bone-homing lines have higher expression of genes required for serine synthesis than MDA-MB-231 parent cells [8, 45], and our flux analysis indeed confirm greater serine synthesis by BoM cells. This has been proposed to facilitate tumor growth by stimulating osteoclast differentiation for bone resorption [4]. The fate of α-ketoglutarate derived from serine synthesis, as well as the majority of oxaloacetate resulting from fatty acid synthesis, is uncertain (and not accounted for in Tables 5 and 6) but of interest given that this could affect the proportion of glycolytic NADH shuttled into the mitochondria (J_6a_) vs. used by other pathways (J_6b_). Virtually all cytoplasmic NADH not used for lactate production was assumed to be shuttled into the matrix for oxidation by the respiratory chain since respiration beyond that required for malic enzyme activity was sufficient to accommodate this flux (accounting for 41% of respiration rate). However, it is possible that cytoplasmic NADH is re-oxidized by MDH1, similar to the scheme proposed for LM cells (see Supplemental Fig. 6). In this case, approximately 68% of cytoplasmic NADH could be re-oxidized by MDH1 while the remaining 32% would be shuttled to the mitochondria for oxidation (i.e., J_6a_ = 0.7 and J_6b_ = 1.5 nmol/min × mg in Table 5). This would modestly affect calculation of pyruvate flux to citrate (increase from 0.5 to 0.8 nmol/min × mg) and the complete oxidation of pyruvate by the TCA cycle (J_12f_, J_14_, J_15_, J_16_ increase from about 0.2 to 0.5 nmol/min × mg).

Despite uncertainty in the fate of cytoplasmic NADH, inhibition of serine synthesis and the associated NADH production with CBR-5884 stimulated glucose uptake (and presumably glycolysis) by both BoM and LM, but not T47D, cells (Fig. 5). NADH production linked to serine synthesis therefore appears to affect the cytoplasmic NADH/NAD ratio sufficiently to exert negative feedback on glycolysis in BoM and LM, but not T47D cells. This is somewhat surprising given that serine-coupled NADH production was not notably higher in BoM and LM cells (9.3, 6.5, and 12.1% of total cytoplasmic NADH was coupled to serine synthesis for T47D, BoM, and LM cells, respectively). The simplest explanation is that BoM and LM glycolytic reactions are more sensitive to changes in NADH/NAD than in T47D cells. A second possibility is that NADH produced in excess of that required by lactate dehydrogenase forms a distinct pool that contributes to regulating glycolysis. In principle, this pool is derived from NADH produced from serine synthesis and from the small glycolytic flux supplying pyruvate to the mitochondria. In this scheme, serine synthesis by BoM and LM cells accounted for 79 and 84% of NADH supplied to this pool, respectively, compared to only 50% by T47D cells (i.e., ~80% of NADH not used by LDH is coupled to serine synthesis in BoM and LM lines). It follows that CBR-5884 would more strongly affect the NADH/NAD ratio of this pool in LM and BoM cells, and hence affect glycolysis. Thus, for tumor cells shunting similar proportions of glucose to serine (3–5% for T47D, BoM, and LM cells), but oxidizing a smaller fraction of pyruvate within mitochondria (e.g., 2–3% of pyruvate for BoM and LM cells vs. 11% for T47D cells), then serine synthesis may be sufficient to exert at least some negative feedback on glycolysis despite its minor contribution to total cytoplasmic NADH.

In summary, lung- and bone-homing lines display distinct metabolic phenotypes, and both differ from the highly metastatic MDA-MB-231 cells from which they were derived. The differences are important in supporting the hypothesis that metabolic “re-wiring” contributes to organ-specific metastasis, and identifying specific metabolic profiles compatible with proliferation of metastatic breast cancers within the lung and bone microenvironments. Furthermore, the profiles highlight metabolic pathways that, if targeted by small molecule therapies, could contribute to treatment of lung- or bone-specific metastasis. High metastatic potential MDA-MB-231 breast cancer cells are metabolically balanced in synthesizing roughly equivalent proportions of ATP from glycolysis and oxidative phosphorylation, but have a high ATP demand, require exogenous serine, and appear to devote considerable resources to combat oxidative stress. Such cells thus appear vulnerable to therapies targeting serine uptake, ATP futile cycles, and antioxidant defense reactions. The lung metastatic line appears to restrict mitochondrial substrate availability, potentially as a redox defense mechanism to limit mitochondrial ROS production in the high oxygen lung microenvironment. Additionally, both the lung- and bone-homing lines synthesize little to no GSH from glutamine and only limited NADPH from the oxidative pentose phosphate pathway, suggesting both may be vulnerable to ROS-generating redox cycling compounds. Lung- and bone-homing lines rely largely on glycolysis for ATP, making this pathway an attractive target. The reverse non-oxidative pentose phosphate pathway, serine synthesis, and NADPH from the folate cycle are also potential targets affecting proliferation of the lung-homing line. It is important to note that the metabolic phenotypes of the lung- and bone-homing lines in this study may not reflect the profiles of all metastatic cancers to these organs. Indeed, more than a single metabolic flux profile is likely conducive for growth in the lung or bone microenvironments. The compilation of profiles from a number of lung- and bone-homing lines is thus necessary to understand what common signatures may exist for organ-specific metastasis.

## Supplementary Information

**Additional file 1. **Flux Equations and Supplemental Figures.

## Data Availability

Data files and models are available to interested parties through the corresponding author (MBJ).
