# Supplementary material for: Breast cancer cells that preferentially metastasize to lung or bone are more glycolytic, synthesize serine at greater rates, and consume less ATP and NADPH than parent MDA-MB-231 cells
Source: Cancer Metab. 2023 Feb 20;11:4. doi: 10.1186/s40170-023-00303-5 (PMC9940388; doi:10.1186/s40170-023-00303-5)
Supplement: Supplementary file 1 — Additional file 1. Flux Equations and Supplemental Figures. [file 40170_2023_303_MOESM1_ESM.docx]

Supplementary Information

The mean fraction of exogenous glutamine used for GSH synthesis (n=3 to 4 experiments per cell line) was determined from the reduction in glutamine uptake (J_10_) with 50 μM BSO referenced to paired controls without BSO. The product of the mean fractional glutamine used for GSH synthesis and J_10_ measured for each experiment with [5-^13^C] glutamine yielded flux to GSH J_18_.

$J_{18}=\left( 1-\frac{J_{10}^{BSO}}{J_{10}^{cntl}} \right)*J_{10}^{13C gln}$ (Eq. 11)

Similarly, the mean fraction of exogenous glutamine used for fatty acid synthesis (n=3-4 experiments per cell line) was determined from the reduction in J_10_ with 40 μM C75 referenced to paired controls without C75. The product of the mean fractional glutamine used for fatty acid synthesis and J_10_ measured for each experiment with [5-^13^C] glutamine yielded J_12r_, the reductive carboxylation of α-ketoglutarate to citrate that contributed to fatty acid synthesis.

$J_{12r}=\left( 1-\frac{J_{10}^{C75}}{J_{10}^{cntl}} \right)*J_{10}^{13C gln}$ (Eq. 12)

The mean fraction of exogenous glucose used for fatty acid synthesis (n=3-4 experiments per cell line) was determined from the decrease in glucose uptake (J_0_) with 40 μM C75 referenced to paired controls without C75. In many experiments, addition of C75 reduced lactate production (J_4_), which was interpreted as a reduction in ATP turnover associated with fatty acid synthesis. As such, C75-sensitive J_0_ was corrected for C75-sensitive J_4_. An additional correction was applied based on the NADPH requirement for palmitate synthesis. For each palmitate synthesized, four glucose are required to generate 8 acetyl-CoA and seven glucose are required to generate 14 NADPH, assuming all NADPH was from the oxidative PPP. Thus, only 36% of C75-sensitive glucose that does not end as lactate is expected to be shunted to fatty acids.

$fraction Glc to FA=0.36*\left[ \frac{{(J}_{0}^{cntl}-J_{0}^{C75})-0.5*(J_{4}^{cntl}-J_{4}^{C75})}{J_{0}^{cntl}} \right]=V$ (Eq. 13)

From the C75 experiments, the rate of citrate flux to fatty acid (J_13_) for experiments with [5-^13^C] glutamine is

$J_{13}=2*J_{0}*V+J_{12r}$ (Eq. 14)

From Model 3 optimized Eq. 8 and 9 (see Methods), the flux from citrate to α-ketoglutarate (J_12f_) in the TCA cycle is

$J_{12f}=\left( \frac{1-W}{W} \right)*X*J_{10}$ (Eq. 15)

From the citrate balance, the rate of pyruvate oxidation (J_5_) is

$J_{5}=J_{13}+J_{12f}-J_{12r}$ (Eq. 16)

From the pyruvate balance and Model 3 optimized Eq. 10, malic enzyme activity (J_7_) is

$J_{7}=Y*(J_{4}+J_{5})$ (Eq. 17)

Flux of α-ketoglutarate to succinyl CoA (J_14_) is

$J_{14}=X*J_{10}+J_{12f}-J_{12r}$ (Eq. 18)

Since J_14_ was assumed to be the only succinyl CoA producer, and J_15_ the only succinyl CoA consumer, J_15_ = J_14_. Malate to oxaloacetate flux (J_16_) is

$J_{16}=J_{15}-J_{7}$ (Eq. 19)

Oxaloacetate derived from glucose-dependent fatty acid synthesis was assumed to return to the TCA cycle to support citrate synthesis by reaction with acetyl CoA.

$J_{17}=J_{16}+2*J_{0}*V$ (Eq. 20)

Glucose consumed by the oxidative PPP (J_1_) and net reverse non-oxidative PPP was defined as that which was not (a) converted to lactate, (b) converted to fatty acid, (c) oxidized by mitochondria, or (d) used for serine biosynthesis. For this study, net J_3r_ (J_3r_ – J_3f_) was expressed as nmol R5P produced/min x mg for direct comparison with J_3f_ expressed as nmol R5P consumed/min x mg, but this expression becomes 5/6*(J_3r_-J_3f_) for glucose equivalents consumed/min x mg.

$J_{1}+{\frac{5}{6}(J}_{3r}-J_{3f})=J_{0}-0.5*J_{4}-V*J_{0}-0.5*\left[ 5*\left( J_{5}-2*J_{0}*V \right)-\left( J_{7}-\frac{J_{4}}{J_{4}+J_{5}}*J_{7} \right) \right]-0.5*J_{19}$ (Eq. 21)

With all expressions on the right side of Eq. 21 known (J_19_ was determined by an iterative process, as detailed below in Eq. 39 and Supplemental Fig. 1), glucose shunted to pentose phosphates for nucleotide synthesis can be calculated. Let ‘A’ represent the numerical result of these expressions to simplify Eq. 21.

$J_{1}+\frac{5}{6}\left( J_{3r}-J_{3f} \right)=A$ (Eq. 22)

Resolving J_1_ and net J_3r_ involved the solutions to Eq. 1 and 2 from Model 1 experiments with [1,2-^13^C] glucose (see Methods). Utilizing Eq. 1 and 2, J_3r_ and J_3f_ (in R5P produced or consumed/min x mg) were calculated for the experiments (n=6) with [1,2-^13^C] glucose as

$J_{3f}=\frac{L}{\frac{2}{3}*(1-L)}*J_{0}$ (Eq. 23)

$J_{3r}=\frac{J_{3f}}{K}$ (Eq. 24)

The mean fractional contribution of net J_3r_ to glucose consumption by the PPP, and the mean ratio of J_3f_ : net J_3r_ in the experiments with [1,2-^13^C] glucose were determined.

$fraction Glc used by net J_{3r}=\frac{{\frac{5}{6}*(J}_{3r}-J_{3f})}{J_{1}+\frac{5}{6}*(J_{3r}-J_{3f)}}=N$ (Eq. 25)

$\frac{J_{3f}}{J_{3r}-J_{3f}}=S$ (Eq. 26)

The results from Eq. 25 were applied to the experiments with [5-^13^C] glutamine to determine net J_3r_ (units of R5P produced/min x mg) by solving for J_1_ in Eq. 22 and substituting this expression in Eq. 25 and rearranging.

$J_{3r}-J_{3f}=\frac{6}{5}*\frac{\left[ \frac{N}{\left( 1-N \right)} \right]*A}{1+[\frac{N}{\left( 1-N \right)}]}$ (Eq. 27)

Forward flux through the non-oxidative PPP (in R5P consumed/min x mg) was calculated from Eq. 26 and 27.

$J_{3f}=S*{(J}_{3r}-J_{3f})$ (Eq. 28)

Reverse flux through the non-oxidative PPP (in R5P produced/min x mg) was calculated from Eq. 1 and Eq. 28.

$J_{3r}=\frac{J_{3f}}{K}$ (Eq. 29)

Hexose phosphate consumption by the oxidative PPP was calculated from Eq. 22.

$J_{1}=A-\frac{5}{6}\left( J_{3r}-J_{3f} \right)$ (Eq. 30)

Ribose phosphate consumption for nucleotide synthesis was calculated by mass balance.

$J_{9}=J_{1}+J_{3r}-J_{3f}$ (Eq. 31)

The conversion of serine to glycine (J_21_) in the folate cycle is coupled to J_9_ by two 10-formyl-tetrahydrofolates required to synthesize purine nucleotides. Using the estimate that 80% of 10-formyl-tetrahydrofolate is derived from serine conversion to glycine in the folate cycle in tumor cells [36] and assuming 50% of J_9_ is for purine nucleotide synthesis, serine flux to glycine in the folate cycle was estimated.

$J_{21}=0.8*J_{9}$ (Eq. 32)

The mean ratio of serine efflux (J_22_) to glucose uptake (J_0_) determined for experiments with [1,2-^13^C] glucose (n=6) was used to calculate J_22_ for experiments with [5-^13^C] glutamine.

$\frac{J_{22}^{13C glc}}{J_{0}^{13C glc}}=B$ (Eq. 33)

$J_{22}={B*J}_{0}^{13C gln}$ (Eq. 34)

The contribution of endogenous glycine to labeling of the glycine pool that can recycle to serine (J_24_) was calculated from Eq. 6 (see Methods).

$J_{24}=\frac{1-Q}{Q}*J_{21}$ (Eq. 35)

The serine mass balance equation (Eq. 36) and Eq. 7 (see Methods) were used to calculate the flux of glycine to serine (J_25_).

$J_{19}+J_{20}+J_{25}=J_{21}+J_{22}$ (Eq. 36)

$J_{25}=\frac{J_{21}+J_{22}}{1+\frac{1}{R}}$ (Eq. 37)

Glycine consumption associated with the folate cycle was calculated from mass-balance.

$J_{23}=J_{21}+ J_{24}-J_{25}$ (Eq. 38)

Serine biosynthesis from 3PG (J_19_) can be calculated from Eq. 5 and 7.

$J_{19}=\frac{P}{R}*J_{25}$ (Eq. 39)

The solution for J_19_ involved an iterative process within Model 3 because of the interdependence of fluxes [J_1_ + (J_3r_ – J_3f_)], J_9_, J_21_, and J_19_ (Supplemental Fig. 1).

Supplemental Figure 1. Iterative process to determining serine biosynthesis flux (J_19_). Flux through the oxidative pentose phosphate pathway (J_1_) and the reverse non-oxidative pentose phosphate pathway (J_3r_) depend in part on J_19_, but J_19_ depends on glycine flux to serine in the folate cycle (J_25_), which in turn depends on serine flux to glycine in the folate cycle (J_21_). J_21_ depends on the flux of pentose phosphates to nucleotides (J_9_), the latter of which is calculated from J_1_. As a result, the first iteration of Model 3 was run with J_19_ set to zero, results in overestimation of glucose consumption by J_1_ and J_3r_. Serine synthesis flux used to calculate glucose consumption by J_1_ and J_3r_ was then manually entered as the model calculated J_19_ from Eq. 39. The process was repeated until the user-entered estimate of J_19_ equaled the model calculated value.


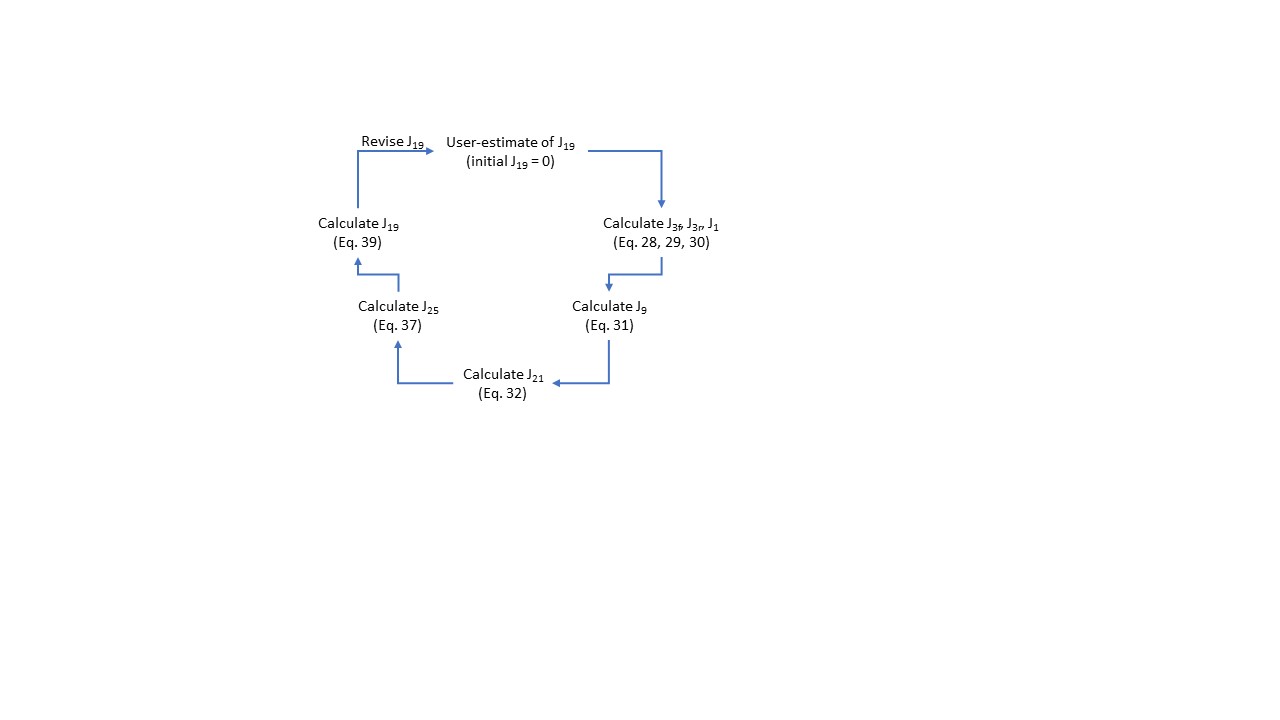


The contribution of endogenous serine to labeling of the serine pool (J_20_) is determined from Eq. 5 (see Methods).

$J_{20}=\frac{1-P}{P}*J_{19}$ (Eq. 40)

Lower glycolytic flux from 3PG to pyruvate (J_2c_) was calculated from mass balance of pyruvate.

$J_{2c}=J_{4}+J_{5}-J_{7}$ (Eq. 41)

Middle glycolytic flux from TrP to 3PG (J_2b_) accounts for J_19_.

$J_{2b}=J_{2c}+J_{19}$ (Eq. 42)

Upper glycolytic flux from HxP to TrP (J_2a_, in units of HxP consumed/min x mg) was calculated from the mass balance of TrP.

$J_{2a}=0.5*(J_{2b}+\frac{1}{3}*J_{3r}-\frac{1}{3}*J_{3f})$ (Eq. 43)

It was assumed that all α-ketoglutarate entering the TCA cycle through J_11a_ and J_11b_ was derived from the consumption of exogenous [5-^13^C] glutamine (J_10_). Glutamine consumption accounted for by J_11a_, J_11b_, and J_18_ was designated as J_10a_, while the remaining unaccounted-for consumption was designated J_10b_.

$J_{10a}=J_{11a}+J_{11b}+J_{18}$ (Eq. 44)

$J_{10b}=J_{10}-J_{10a}$ (Eq. 45)

One important constraint of Model 3 (and Model 1) was that the calculated mitochondrial respiration rate, based on reducing equivalents generated by pyruvate dehydrogenase, the TCA cycle, and glycolysis, had to equal the measured rate. The stoichiometry of mitochondrial reducing equivalents from pyruvate oxidation was constant, but that from glutamine consumption coupled to malate production for J_7_ and citrate production for fatty acid synthesis (via J_12r_) could vary, depending on the fraction of α-ketoglutarate supplied by glutamate dehydrogenase (J_11a_) vs. serine synthesis (J_11b_). Three reducing equivalents are coupled with α-ketoglutarate from J_11a_ and its subsequent conversion to malate in the TCA cycle, whereas only two mitochondrial reducing equivalents are coupled to J_11b_ since α-ketoglutarate production by J_19_ yields one cytoplasmic reducing equivalent. Similarly, one mitochondrial reducing equivalent is coupled to α-ketoglutarate supply by J_11a_ that is reductively carboxylated to citrate for fatty acid synthesis, whereas no mitochondrial reducing equivalents are coupled to J_12r_ if α-ketoglutarate is supplied by J_11b_.

$Reducing equivalent stoich for J_{7}=3*\left( \frac{J_{11a}}{J_{11a}+J_{11b}} \right)+2*\left( \frac{J_{11b}}{J_{11a}+J_{11b}} \right)=C$ (Eq. 46)

$Reducing equivalent stoich for J_{12r}=\frac{J_{11a}}{J_{7}}=D$ (Eq. 47)

The default condition assumed was that J_11a_ (mitochondrial oxidation of glutamate to α-ketoglutarate) supplied all α-ketoglutarate for both J_7_ and J_12r_ (i.e., J_11b_ = 0 and J_11a_ = J_7_ + J_12r_), and all cytoplasmic NADH from glycolysis and serine synthesis was oxidized by mitochondria (i.e., J_6b_ = 0 and J_6a_ = J_2b_ + J_19_ – J_4_). Equations 48 and 49 reflect the supply rates of α-ketoglutarate to the TCA cycle and cytoplasmic NADH for reactions other than lactate dehydrogenase (J_4_).

$J_{11a}+J_{11b}=J_{7}+J_{12r}$ (Eq. 48)

$J_{6a}+J_{6b}=J_{2b}+J_{19}-J_{4}$ (Eq. 49)

The contribution of J_11b_ to α-ketoglutarate supply to the TCA cycle was considered only when J_7_ was not adequately optimized to achieve the absolute minimal error in predicting the ^13^C lactate labeling pattern because of the constraint imposed by the measured respiration rate. This occurred for LM cells in spite of optimizing Eq. 11 to Z=0 (i.e., none of the cytoplasmic NADH oxidized by mitochondria) because this cell line had a very low respiration rate. J_11b_ was increased until the minimum error was reached, or J_11b_ = J_19_, or J_11a_ = 0. Thus, the calculation of respiration rate was

$J_{RR}^{calc}=2*\left( J_{0}*V \right)+5*\left[ J_{5}-2*\left( J_{0}*V \right) \right]+Z*\left( J_{2b}+J_{19}-J_{4} \right)+C*J_{7}+D*J_{12r}$ (Eq. 50)

Cellular NADPH consumption was calculated from the reactions producing NADPH.

$J_{8}=2*J_{1}+J_{7}+J_{21}-J_{25}$ (Eq. 51)

Mitochondrial ATP synthesis rate was calculated from the stoichiometries of 10 H^+^ pumped by the respiratory chain for each matrix-derived NADH oxidized, 6 H^+^ pumped for each matrix-derived FADH_2_ oxidized, 9 net H^+^ pumped for each cytoplasmic-derived NADH oxidized, and 11 net H^+^ returned to the matrix for three ATP synthesized by ATP synthase and subsequently exported to the cytoplasm [10], together with the fraction of oligomycin-sensitive mitochondrial respiration:

$\frac{\left( 10*J_{5}+10*J_{12f}+9*J_{14}+6*J_{15}+10*J_{16}+10*J_{11a}+9*J_{6a} \right)*\frac{{(J}_{RR}-J_{oligoRR})}{J_{RR}}}{\frac{11 H+}{3 ATP}}+J_{14}$ (Eq. 52)

Glycolytic ATP synthesis rate, including J_0_, was calculated as:

$J_{2b}+J_{2c}-J_{0}-J_{2a}$ (Eq. 53)

Supplemental Figure 2


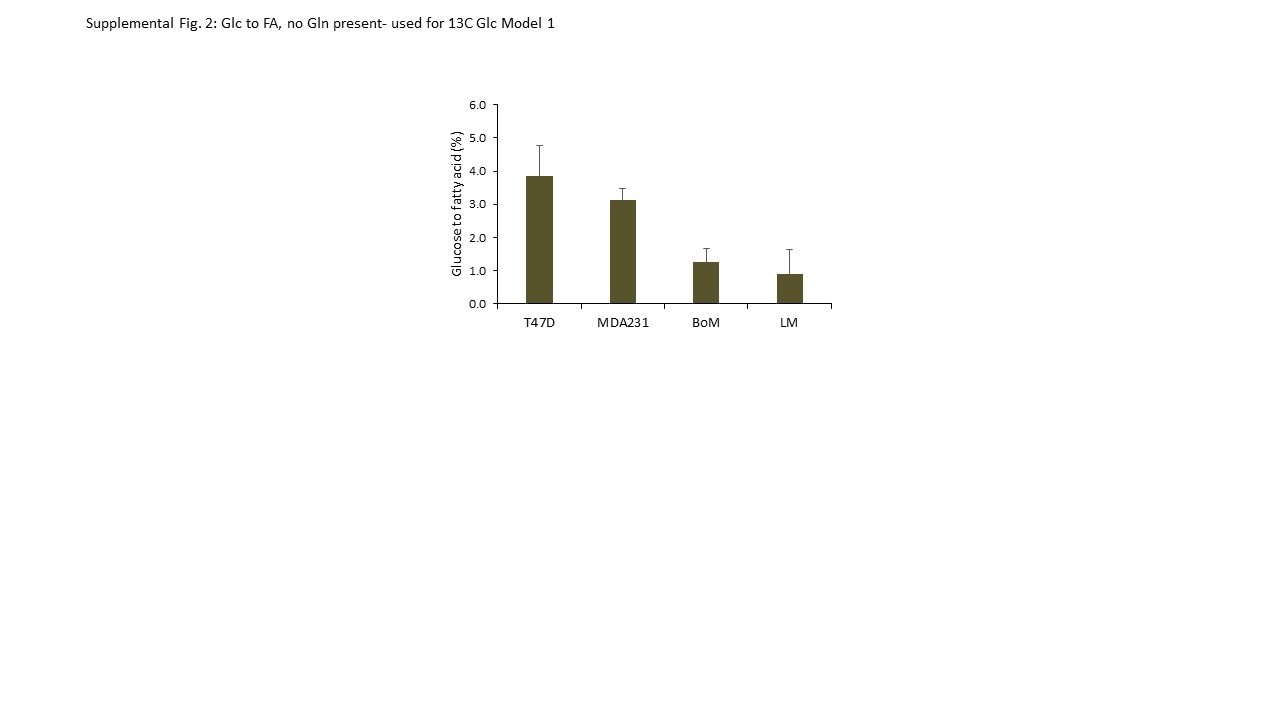


Supplemental Figure 2. *De novo* synthesis of fatty acids from glucose for cells metabolizing exogenous glucose but not glutamine. Cells were pre-treated 3-4 h with 40 μM C75 or equivalent volume of vehicle (100% ethanol) to inhibit fatty acid synthesis. The decrease in glucose consumption relative to controls was used to assess flux of glucose to fatty acids as detailed in Supplemental Information Eq. 13. From one-way ANOVA, there was a significant main effect of cell line (p = 0.033), but no significant differences from Tukey’s post-hoc test (p = 0.050 for T47D vs. LM cells).

Supplemental Figure 3

Supplemental Figure 3. Relationship of anabolic and bioenergetic properties to cell growth. (A) Bi-plot of Scores and Loadings from principal component analysis of anabolic and bioenergetic indices. The percentage of glucose consumed that was shunted to nucleotides (NTs), serine (Ser), and fatty acids (FA), the percentage of glutamine shunted to FA and glutathione (GSH), and the percentage of pentose phosphates (R5P) from the reverse non-oxidative PPP (NonPPP) were the anabolic indices used. The percentage of ATP derived from oxidative phosphorylation was the bioenergetic index included. (B) Cell line proliferation rate in cell culture media estimated in the 3-7 days preceding experiments with [5-^13^C] glutamine. Cells were seeded in 10 cm dishes at 1-2 x 10^6^ cells (T47D), 0.5-0.6 x 10^6^ cells (MDA-MB-231, BoM), or 0.8-1.0 x 10^6^ cells (LM) then counted after 3 (BoM, LM), 4-5 (MDA-MB-231), or 5-7 (T47D) days to calculate average proliferation rate over the culture time. Data are mean ± SEM of four experiments. Bars sharing common letters are not significantly different. (C) Principal component regression analysis of anabolic and bioenergetic indices to proliferation rate.


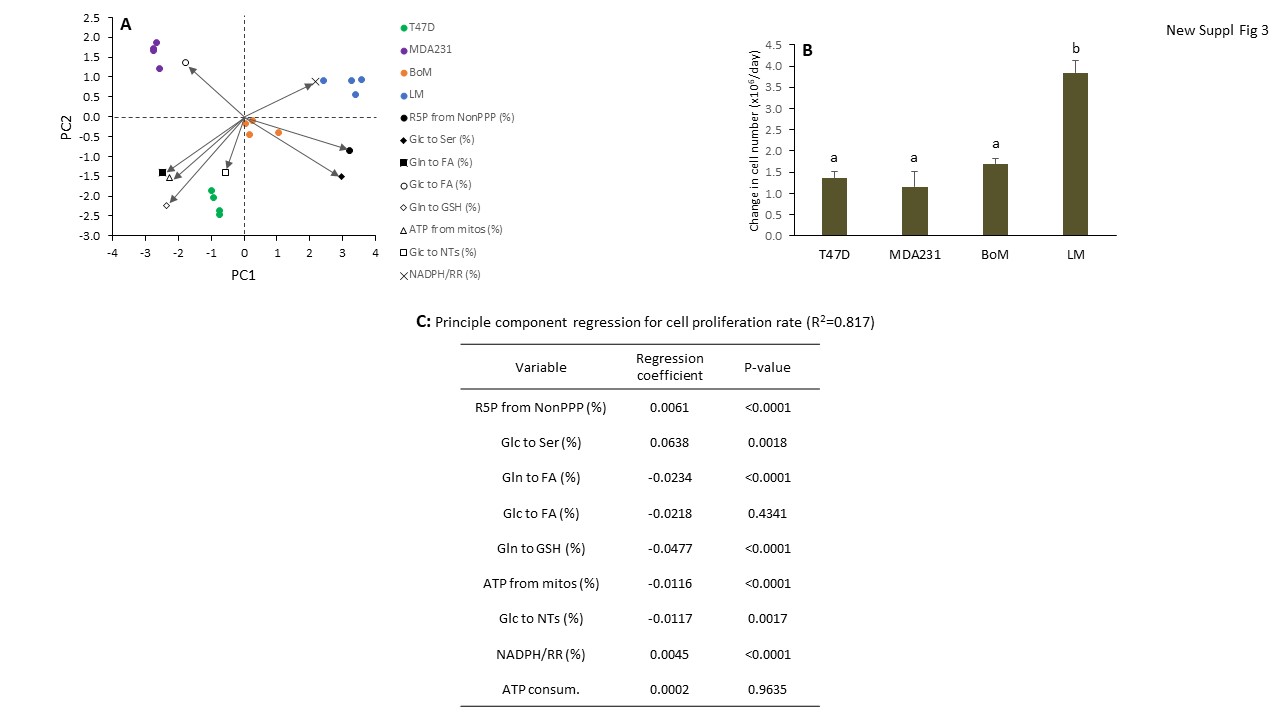


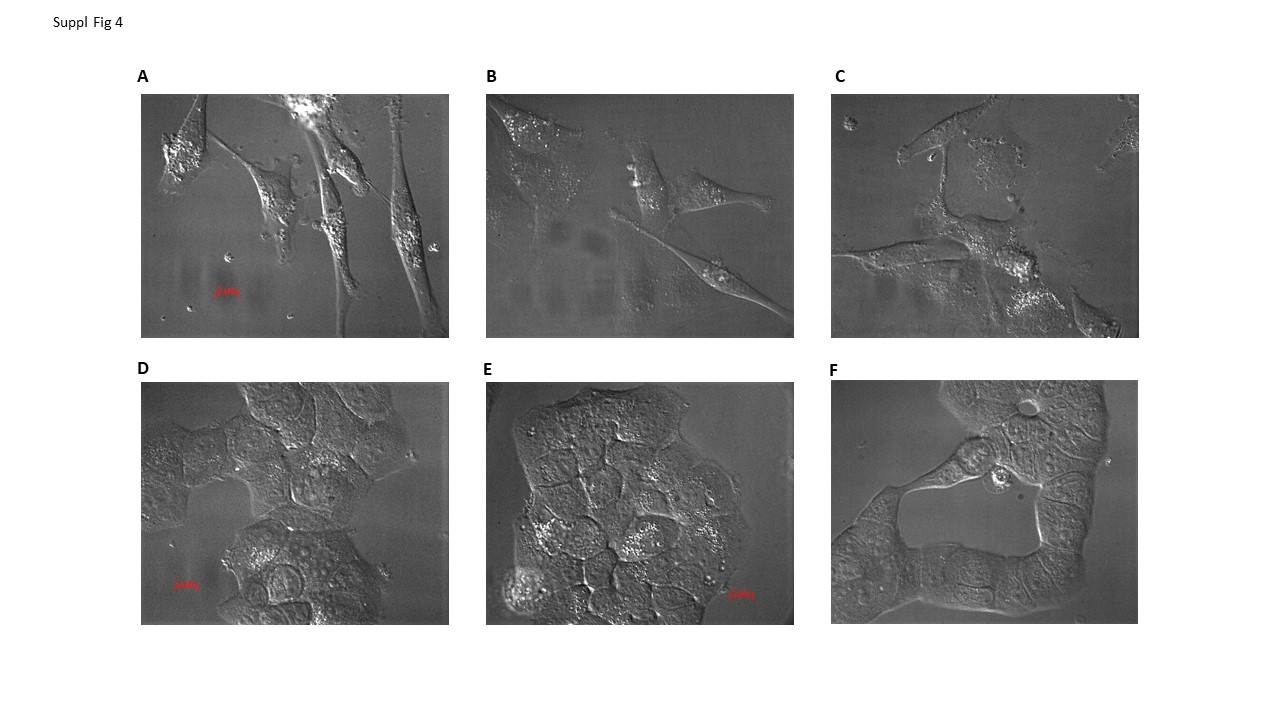
Supplemental Figure 4

Supplemental Figure 4. Morphology of MDA-MB-231 and T47D cells. Representative images of MDA-MB-231 cells (A-C) and T47D cells (D-F) cultured with experimental buffer in 2-well Lab-Tek chambers for flux analysis. Images were acquired with a 63x oil objective on a Zeiss 200M Axiovert microscope. Red scale bar is 5 μm.

Supplemental Figure 5

Supplemental Figure 5. MDA-MB-231 proliferation rate is lower than predicted from the relative rate of NADPH used for biosynthesis. NADPH consumption normalized to respiration rate is an index of that used for biosynthesis assuming that mitochondrial ROS production directly correlates with respiration rate. The regression line was from analysis of T47D, BoM, and LM lines, with MDA-MB-231 cells being an outlier. Data are mean ± SEM of four experiments for each cell line. The high NADPH/respiration rate ratio of MDA-MB-231 cells relative to their low proliferation rate suggests a higher proportion of NADPH is used for protection from ROS rather than biosynthesis.


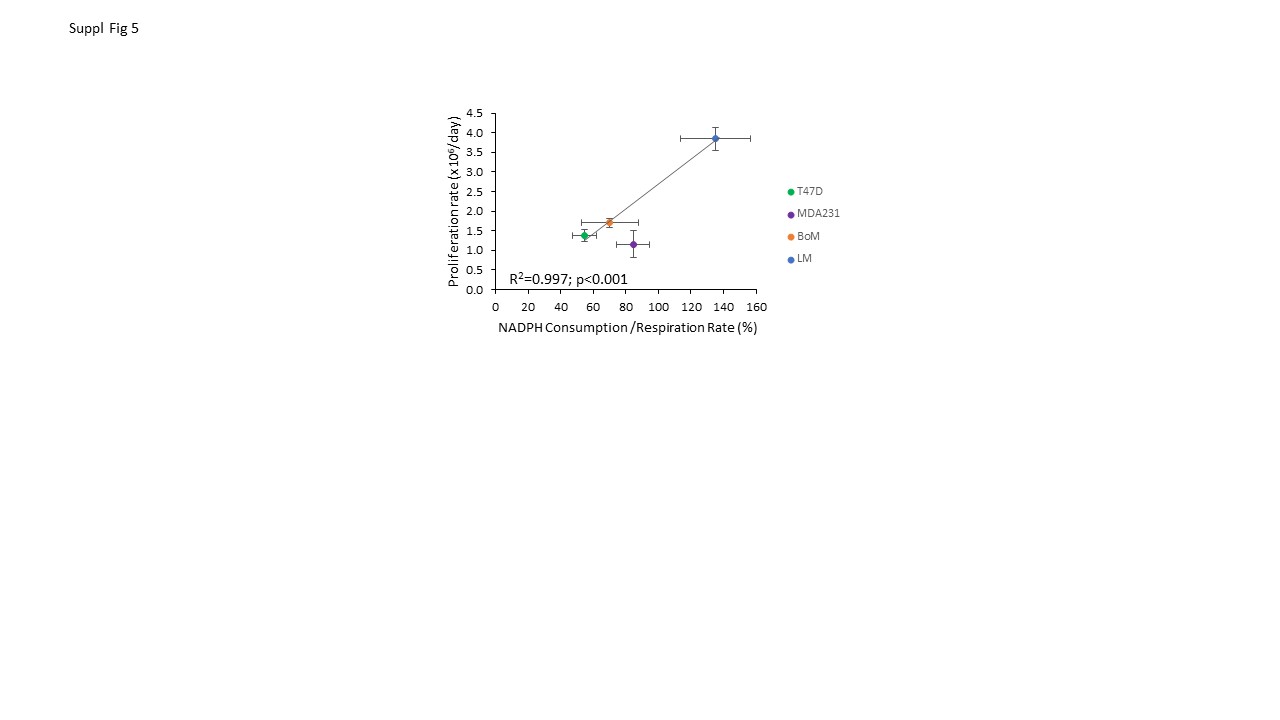


Supplemental Figure 6


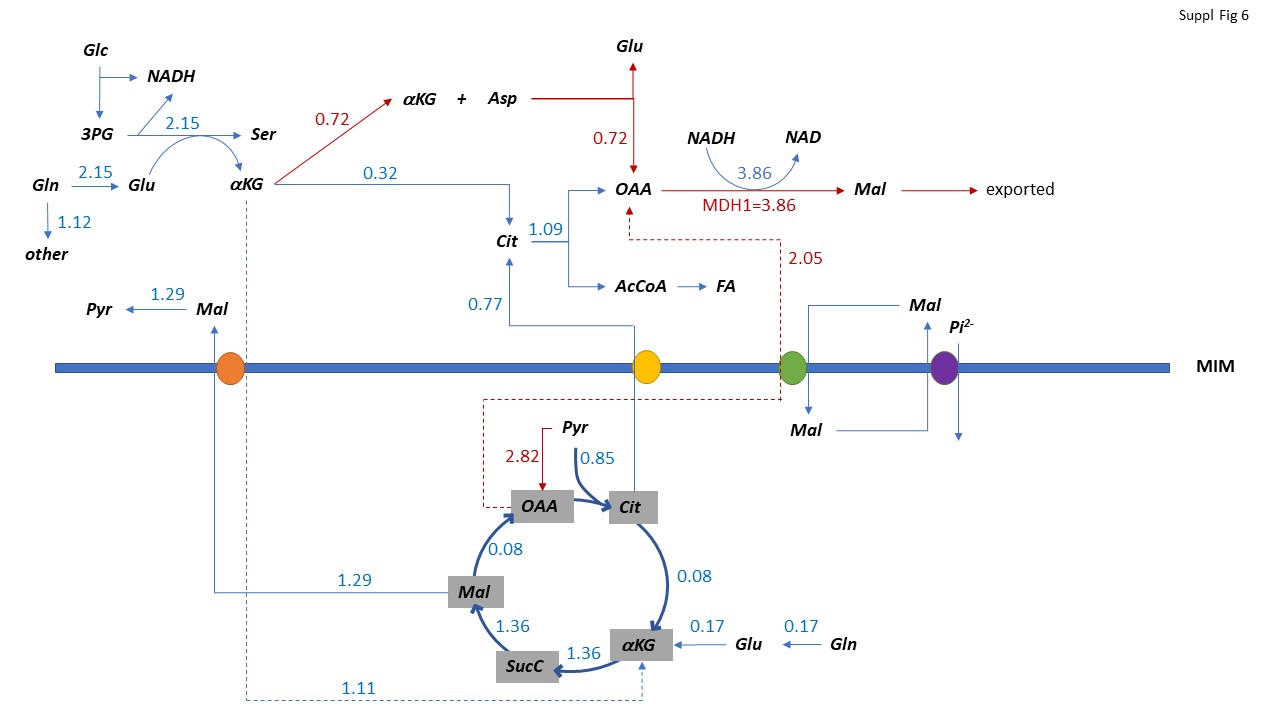


Supplemental Figure 6. Detailed LM flux scheme assuming cytoplasmic NADH not used by lactate dehydrogenase is re-oxidized by malate dehydrogenase 1 (MDH1). Measured LM fluxes from Tables 5 and 6 are shown as blue numbers with blue arrows. LM cells generate 3.86 nmol cytoplasmic NADH/min above that required for lactate production and limited mitochondrial respiration rate precludes its oxidation by mitochondria through the malate-aspartate shuttle, suggesting this shuttle is not operating in these cells. If MDH1 re-oxidizes the ‘excess’ NADH, then fatty acid synthesis and residual α-ketoglutarate from serine synthesis can supply only 47% of the required oxaloacetate (OAA) to MDH1. The remaining OAA (2.05 nmol/min) is proposed to come from pyruvate carboxylase, which has previously been shown to be upregulated in some lung-homing lines. Red arrows indicate hypothetical fluxes not shown in Tables 5 or 6. Thick blue line is the mitochondrial inner membrane (MIM), and proposed carriers involved are shown as circles. Orange circle: α-ketoglutarate carrier, yellow circle: tricarboxylate carrier, green circle: OAA carrier, and purple circle: dicarboxylate carrier.
